# *In situ* reprogramming of fibroblasts into antigen-presenting pseudo-dendritic cells via IFN-β-engineered protoplast-derived exosomes delivered by microneedle arrays to enhance adaptive immunity

**DOI:** 10.7150/thno.115080

**Published:** 2025-08-16

**Authors:** Yue Yin, Shijie Zhao, Wei Li, Yuan Cui, Thanh Loc Nguyen, Ge Gao

**Affiliations:** 1 Advanced Technology Research Institute, Beijing Institute of Technology, Jinan, 250307, China.; 2 School of Medical Technology, Beijing Institute of Technology, Beijing, 100081, China.; 3 South Australian immunoGENomics Cancer Institute (SAiGENCI), Faculty of Health and Medical Sciences, The University of Adelaide, Adelaide, South Australia 5005, Australia.

**Keywords:** exosome-loaded microneedle arrays, fibroblast, dendritic cell, *in situ* reprogramming, adaptive immunity

## Abstract

**Rationale**: Dendritic cells (DCs) play a crucial role in adaptive immune responses; however, *ex vivo* differentiation strategies face operational complexities and reduced cellular viability. *In situ* reprogramming of resident cells into antigen-presenting cells represents a promising alternative approach for enhancing local immune responses.

**Methods**: We initially introduce the novel concept of pseudo-DCs, *in situ* transforming intradermal fibroblasts into DC-like cells using an engineered exosome-loaded microneedle (MN) array. Specifically, engineered nano-protoplasts expressing interferon-beta (IFN-β) and loaded with varicella-zoster virus glycoprotein E (VZV gE) were used to stimulate DCs and derive immunostimulatory exosomes. These exosomes were integrated into a microarray-based delivery system for intradermal application.

**Results**: The engineered exosomes (IdE@E) induced resident fibroblasts to upregulate DC surface co-stimulatory markers (CD80/86) and effectively present the model antigen. Transcriptome analysis also revealed significant upregulation of genes associated with immune response and antigen presentation in IdE@E-treated cells. *In vivo* studies demonstrated that MN array-delivered IdE@E effectively induced the expression of DC and activation markers from fibroblasts in dermis. Furthermore, MN array-delivered IdE@E significantly elevated the population of IFN-γ^+^CD8^+^ T cells in both lymph nodes and spleen, indicating enhanced local and systemic immune responses.

**Conclusions**: This novel *in situ* reprogramming method represents a paradigm shift in precision immunotherapies, leveraging exosome-mediated cellular mimicry to enhance adaptive immunity without complete cellular transformation. This scalable framework holds significant promise for immunotherapy and could revolutionize personalized immunotherapy.

## Introduction

Dendritic cells (DCs) play a pivotal role in bridging innate and adaptive immunity through their unique ability to process antigens, express co-stimulatory molecules (e.g., CD80/86), and prime T-cell responses 1-3. While conventional DCs originate from committed bone marrow progenitors 4, 5, recent research has focused on reprogramming stem cells and somatic cells (e.g., fibroblasts) to mimic antigen-presenting features 6-9. For instance, Sontag et al. demonstrated the direct reprogramming of mouse and human fibroblasts into DCs using defined transcription factors 10. Similarly, Sachamitr et al. showed that human induced pluripotent stem cells could be differentiated into functional DCs capable of activating T cells 11. Despite these advances, current *ex vivo* differentiation and reintroduction strategies face significant challenges, including operational complexities and reduced cell viability 12-14, necessitating innovative *in situ* strategies.

To address these limitations, research has shifted toward innovative *in situ* strategies, particularly the reprogramming of other kinds of cells into DC-like cells as a prophylactic approach in healthy individuals. This shift is supported by emerging evidence suggesting that partial phenotypic reprogramming may be sufficient to elicit T-cell activation 15. Such a strategy could circumvent the need for *ex vivo* manipulation and complete cellular reprogramming while leveraging endogenous cellular reservoirs to enhance adaptive immunity 16-18. Exosomes have emerged as key mediators of intercellular communication in biomedical research 19, 20. However, their applications have largely been limited to the passive delivery of biomolecules, with their potential to induce cellular phenotype changes remaining underexplored 21, 22. A critical aspect of exosome functionality is their capacity for membrane fusion with target cells 23-25. This process not only facilitates cargo transfer but may also directly modify recipient cell phenotypes and surface molecule expression. By leveraging this capability, exosome-based strategies could induce specific phenotypic changes in recipient cells, potentially including antigen-presenting pseudo-DCs 26, 27. This innovative approach introduces the concept of pseudo-DCs - partially reprogrammed native cells that exhibit key antigen-presenting capabilities without undergoing complete cellular transformation. This strategy aims to harness the immunostimulatory potential of resident cells, offering a more flexible and efficient method to enhance adaptive immune responses *in situ*.

A critical consideration in this context is the selection of an appropriate and accessible anatomical site that facilitates the concentrated application of these exosomes. Such a site should enable the localized reprogramming of recipient cells, thereby maximizing their efficacy while avoiding indiscriminate distribution throughout the body. This targeted approach is essential for optimizing the inducing process and subsequent cellular activation for T cells. The selection of an accessible anatomical site is crucial for the concentrated application of exosomes to maximize efficacy and avoid systemic distribution. Antiviral vaccine development faces challenges from antigenic variability, suboptimal antigen presentation, limited cellular immunity, and viral immune evasion mechanisms, such as major histocompatibility complex (MHC) downregulation 28. Many traditional vaccine platforms, such as inactivated or subunit vaccines, often induce weaker T cell responses compared to modern platforms and can face safety or delivery challenges 29, 30. Among all body regions, the skin stands out as an ideal target owing to its rich fibroblast content and suitability for non-invasive interventions 31, 32, enabling localized T cell activation while minimizing systemic distribution and maximizing efficacy. Fibroblasts, traditionally regarded as structural support cells, have recently been recognized for their capacity to modulate immune responses by secreting cytokines and chemokines that orchestrate immune cell recruitment and activation during infection 33. By harnessing this immunomodulatory potential, the *in situ* reprogramming of fibroblasts into antigen-presenting pseudo-DCs offers a novel strategy to transform a local stromal population into a potent initiator of protective immunity. Additionally, current research has yet to utilize existing intradermal delivery technologies to achieve this specific reprogramming process in fibroblasts 34-36.

**Figure 1:**
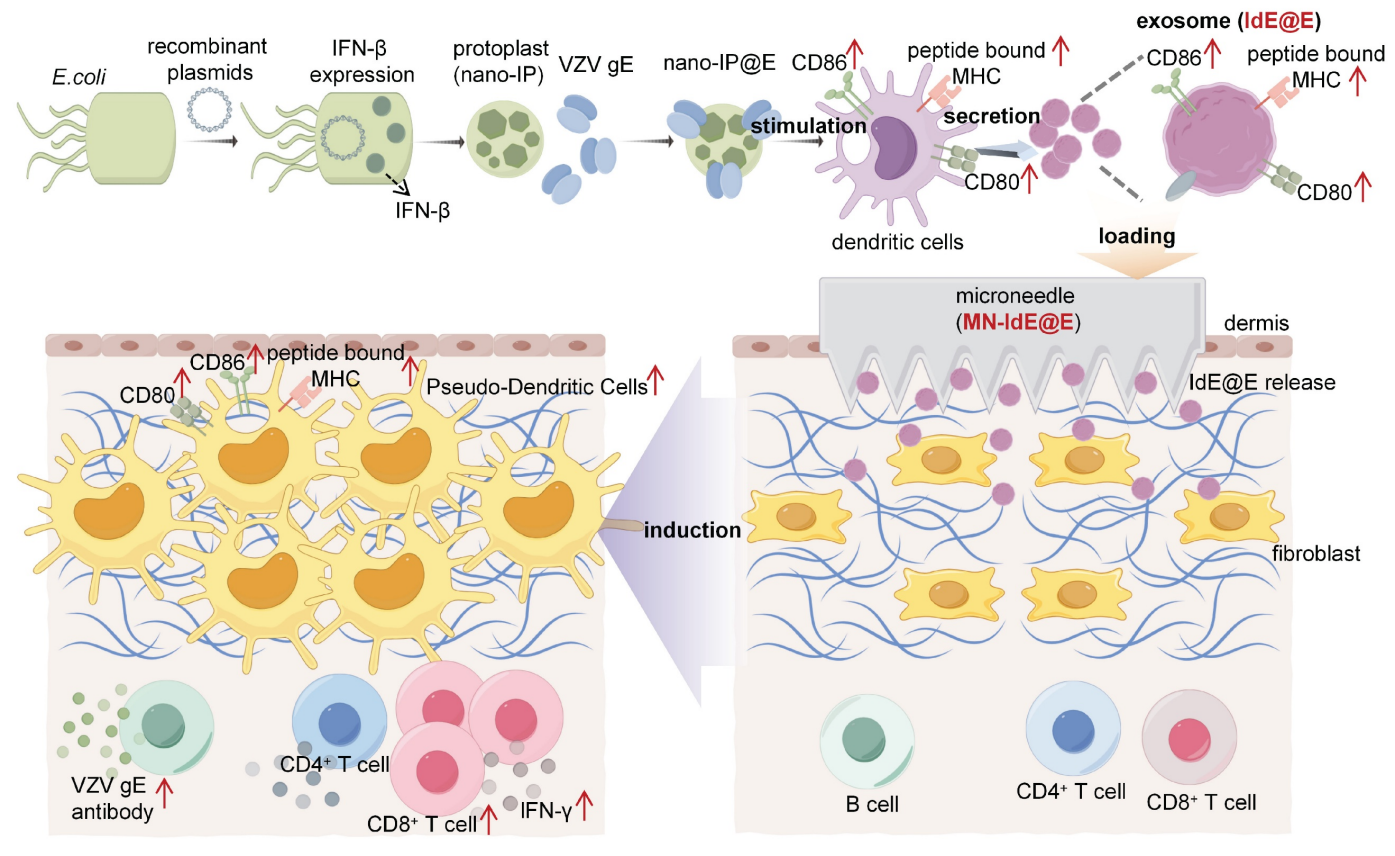
** Schematic illustration of the exosome-loaded MN array system for *in situ* reprogramming of antigen-presenting pseudo-DCs from fibroblasts.** This study presents an innovative exosome-loaded MN array system designed for *in situ* reprogramming of antigen-presenting pseudo-DCs from fibroblasts to enhance adaptive immune responses. The system employs recombinant plasmids to express IFN-β in *E. coli*, which is then combined with VZV gE to form nano-IP@E complexes. These complexes stimulate DCs to produce highly immunogenic exosomes, IdE@E, which are subsequently loaded into dissolvable MN arrays. Upon transdermal administration, IdE@E are released into the dermis, inducing DC-like features in skin fibroblasts, including upregulation of CD80, CD86, and peptide-bound MHC expression. These antigen-presenting pseudo-DCs then activate antigen-specific T cells, promoting cytokine production (e.g., IFN-γ) and stimulating B cells to produce VZV gE-specific antibodies. By leveraging the abundant fibroblast resources in the skin, this approach achieves localized enhancement of adaptive immune responses, providing an innovative platform for developing novel immunomodulatory strategies. Schematic diagram was created using Figdraw.

To address these challenges, we developed an exosome-loaded microneedle (MN) array system designed to induce antigen-presenting pseudo-DCs from fibroblasts *in situ*, focusing on key DC phenotypic characteristics rather than complete cellular reprogramming, enhancing MHC presentation and stimulating robust CD4⁺ and CD8⁺ T cell responses in preclinical models. The choice of MN technology was crucial for our approach, offering precise intradermal delivery, enhanced local immune responses, and improved patient compliance. This MN-based approach enables targeted delivery to the dermis, rich in fibroblasts, while minimizing systemic distribution 37-40. We engineered *Escherichia coli* (*E. coli*) nano-protoplasts expressing IFN-β (nano-IP) and loaded them with a model antigen, varicella-zoster virus glycoprotein E (VZV gE), abbreviated as nano-IP@E. These nano-protoplasts were used to stimulate DCs and derive immunostimulatory exosomes (IdE@E). When integrated into a MN array-based delivery system and applied intradermally, these exosomes induced resident fibroblasts to upregulate DC surface co-stimulatory markers (CD80/86) and effectively present the model antigen (**Figure 1**). This approach achieves rapid, localized immune priming with minimal invasiveness, bypassing the need for endogenous DC intermediation and complete cellular transdifferentiation. Moreover, the modular design of this experimental platform allows rapid incorporation of antigens, such as VZV gE used in existing vaccines, enabling potential adaptability to emerging viral variants. Our novel *in situ* reprogramming method represents a paradigm shift in precision immunotherapies, leveraging strategic cellular mimicry to enhance adaptive immunity. This scalable framework holds significant promise for prophylactic applications in healthy individuals, particularly in viral infection prevention, by enabling the development of personalized, targeted, and efficient immune responses. The potential impact of this approach extends beyond immediate applications, potentially revolutionizing our approach to vaccine development and personalized immunotherapy.

## Methods

### Materials

The plasmid pET-22b and *E. coli* BL21(DE3) competent cells were purchased from ABIOCENTER (Beijing, China). Isopropyl β-D-1-thiogalactopyranoside (IPTG), Ethylenediaminetetraacetic acid (EDTA), sucrose, β-mercaptoethanol, and pepsin were purchased from Sigma-Aldrich (St. Louis, MO, USA). Porcine-derived type-I collagen (I-Col) and sodium hydroxide were purchased from Aladdin (Shanghai, China). Polyvinyl alcohol (PVA, MW 124k) was purchased from Bjoka (Beijing, China). VZV gE was purchased from ACROBiosystems (Beijing, China). Roswell Park Memorial Institute (RPMI) 1640 medium, Eagle's Minimal Essential Medium (DMEM) medium, penicillin/streptomycin, fetal bovine serum (FBS), and trypsin-EDTA were purchased from Gibco (Waltham, MA, USA). Lipopolysaccharide (LPS), LB medium, lysozyme, sucrose, Cell Counting Kit-8 (CCK-8) cell viability assay kit, phosphate-buffered saline (PBS), and fluorescein isothiocyanate (FITC) were purchased from Solarbio (Beijing, China). Lysotracker red, 4′,6-diamidino-2-phenylindole (DAPI), 1,1'-dioctadecyl-3,3,3',3'-tetramethylindocarbocyanine perchlorate (DiI), and 3,3'-dioctadecyloxacarbocyanine perchlorate (DiO) were purchased from Beyotime (Shanghai, China). The BCA protein assay kit and exosome extraction kit were from Thermo Fisher Scientific (Waltham, MA, USA). All antibodies used for flow cytometry and other flow cytometry reagents were purchased from Biolegend (San Diego, CA, USA), including anti-mouse CD16/CD32, CD11c, CD80, CD86, CD3, CD4, CD8, IFN-γ, CD44, CD62L, CD19, and CD138 antibodies. Antibodies used for immunofluorescence and Western blot, including anti-CD11c, CD86, and CD80, were obtained from Servicebio (Wuhan, China). The CD63, CD80, CD11c, MHC-II, and IL-6 detection ELISA kits were purchased from Shanghai Yuanmu Biological Technology Co., Ltd.(Shanghai, China). L929 fibroblast cell line and DC2.4 cell line were obtained from the American Type Culture Collection (ATCC, Manassas, VA, USA). The 3D bioprinter was acquired from Cellink (Gothenburg, Sweden). Experimental C57BL/6 mice were purchased from SPF Biotechnology Co., Ltd.(Beijing, China). All other chemical reagents not specifically mentioned were of analytical grade.

### Cloning and expression of IFN-β gene

The mouse IFN-β gene sequence was inserted into the pET-22b(+) expression vector using standard molecular cloning techniques. The recombinant plasmid was transformed into competent *E. coli* BL21(DE3) cells using the heat shock method. Positive clones were selected and cultured overnight in LB medium containing ampicillin (100 μg/mL). The next day, the bacterial culture was diluted 1:100 in fresh LB medium and grown at 37 °C until the OD600 reached 0.6-0.8. IPTG was added to a final concentration of 0.5 mM to induce IFN-β expression, and the culture was incubated at 16 °C for 16 h.

### Preparation of nano-protoplasts and fusion with VZV gE

Induced bacterial cells were harvested by centrifugation at 4,000 rpm for 15 min at 4 °C. The cell pellet was washed twice with ice-cold PBS and resuspended in a buffer containing lysozyme (1 mg/mL) and 20% sucrose. The suspension was incubated at 37 °C for 1 h with gentle shaking. Protoplasts were collected by centrifugation at 3,000 g for 10 min at 4 °C. The protoplast suspension was placed in an ice bath and subjected to ultrasonic treatment using a probe sonicator (200 W power, 30% amplitude, 5 s on/5 s off cycles, for a total of 5 min). The resulting nano-protoplast particles were collected by ultracentrifugation at 100,000 g for 1 h at 4 °C and resuspended in sterile PBS. VZV gE protein (1 mg/mL) was mixed with nano-protoplasts (10 mg/mL) in a 1:1 volume ratio. The entire fusion process was carried out at 4 °C to protect the activity of VZV gE. The mixture was sonicated using a probe sonicator (200 W power, 30% amplitude, 3 s on/2 s off cycles for a total of 10 min) and incubated for 2 h with gentle rotation. The fused nano-IP@E was collected by ultracentrifugation at 100,000 g for 1 h at 4 °C and resuspended in sterile PBS. The protein concentration was determined using a BCA protein assay kit.

### Characterization of nano-IP@E

The size distribution and polydispersity index (PDI) of nano-IP and nano-IP@E were measured using dynamic light scattering (DLS) with a Zetasizer Nano ZS (Malvern Instruments). Zeta potential measurements were also performed using the same instrument. All measurements were conducted at 25 °C. SDS-PAGE analysis was performed to characterize the protein composition of nano-IP@E and nano-P. ELISA was conducted to quantify IFN-β and VZV gE expression in control, nano-P, nano-IP, and nano-IP@E samples.

### Evaluation of nano-IP@E-induced cytotoxicity in DCs

The potential cytotoxic effects of nano-IP@E on DC2.4 cells were examined using a CCK-8 viability assay. Cells were seeded at a density of 1 × 10^4^ cells per well in 96-well plates and allowed to adhere overnight. The following day, cells were exposed to varying concentrations (0-20 μg/mL) of the nanoparticles for 48 h. Following incubation, the CCK-8 reagent was introduced, and absorbance measurements at 450 nm were obtained via a microplate reader. Cell viability was determined relative to untreated controls, which were assigned 100% viability.

### Cellular uptake of nano-IP@E by DCs

FITC-labeled nano-IP@E uptake and intracellular trafficking in DC2.4 cells were analyzed using a confocal microscope (ZEISS LSM880). DC2.4 cells were seeded at a density of 2 × 10^5^ cells per well in 35 mm glass-bottom dishes and cultured overnight. The following day, cells were incubated with these fluorescent nanoparticles for 4 h. Prior to imaging, nuclei and lysosomes were stained with specific markers. Post-incubation, the cells were fixed and observed using appropriate laser settings to visualize FITC, nuclear, and lysosomal signals.

### DCs activation induced by nano-IP@E

DC2.4 cells were seeded at a density of 2 × 10^5^ cells per well in 6-well plates (1 × 10^5^ cells/mL) and cultured overnight. The next day, DC activation was assessed after 24 h exposure to various treatments: 20 μg/mL of nano-P (from normal *E. coli*), nano-IP, nano-IP@E, or 1 μg/mL of LPS. Cells were then harvested, washed with cold PBS, and incubated with fluorochrome-conjugated antibodies targeting CD86, CD80, and MHC-II for 30 min at 4 °C in darkness. Following incubation, the cells were resuspended in FACS buffer and analyzed via a flow cytometer (BD FACSAria™III Cell Sorter), and data were analyzed using FlowJo software (FlowJo™ v10.10).

### Extraction of IdE@E

DC2.4 cells were seeded at a density of 1 × 10^5^ cells/mL. Nano-IP@E was added to the culture at a final concentration of 20 μg/mL. The cells were incubated at 37 °C in a 5% CO_2_ atmosphere for 48 h. The culture supernatant was collected and subjected to centrifugation to remove cells and debris. The supernatant was ultracentrifuged at 100,000 × g for 60 min to pellet exosomes after using an exosome extraction kit. The exosome pellet was washed once with sterile PBS and resuspended in a small volume of PBS. The protein concentration was determined using a BCA protein assay kit, and the exosomes were stored at -80 °C until use.

### Characterization of IdE@E

The morphology of dEV and IdE@E was examined using transmission electron microscopy (TEM). Size distribution, PDI, and zeta potential were measured using DLS. DNA and RNA content were analyzed using a NanoDrop spectrophotometer (Thermo Fisher Scientific). Protein markers (CD63, CD80, CD11c, and MHC-II) were first analyzed via commercial ELISA kits according to the manufacturer's instructions. Protein markers (CD11c, CD80, CD86, and MHC-II) were analyzed via magnetic bead-based detection. Briefly, streptavidin-coated magnetic beads were conjugated with biotinylated antibodies against exosomal surface antigens. Exosomes were incubated with these beads, and the resulting complexes were analyzed via a flow cytometer (BD FACSAria™III Cell Sorter), and data were analyzed using FlowJo software (FlowJo™ v10.10). The key marker CD80 was further analyzed via Western blot.

### Cellular uptake of IdE@E by L929 cells

L929 cells were seeded at a density of 2 × 10⁵ cells per well in 35 mm glass-bottom dishes and cultured overnight. The following day, cells were incubated with DiO-labeled dEV or IdE@E (green) for 4 h, then stained with LysoTracker Red for lysosomes and DAPI for nuclei. For membrane fusion studies, cells were additionally labeled with DiI. Images were acquired using a confocal laser scanning microscope (ZEISS LSM880).

### Analysis of cell reprogramming

L929 cells or primary mouse skin fibroblasts were cultured in RPMI 1640 medium, respectively, both supplemented with 10% FBS and 1% penicillin-streptomycin. For experiments, cells were seeded at a density of 5 × 10^4^ cells/mL. After 24 h of cell attachment, IdE@E was added to the culture medium at a final concentration of 50 μg/mL. Cells were incubated for an additional 24 h under standard culture conditions. Cells were harvested, washed with PBS, and resuspended in FACS buffer. Fc receptors were blocked with anti-mouse CD16/CD32 antibody for 15 min at 4 °C. Cells were then stained with fluorochrome-conjugated antibodies against CD80, CD86, MHC-II, and CD11c and analyzed via a flow cytometer (BD FACSAria™III Cell Sorter), and data were analyzed using FlowJo software (FlowJo™ v10.10).

### Transcriptome sequencing

Total RNA was extracted from treated and untreated cells using TRIzol reagent according to the manufacturer's instructions. RNA quality and quantity were assessed using an Agilent 2100 Bioanalyzer. Library preparation was performed using the TruSeq Stranded mRNA Library Prep Kit. Sequencing was carried out on an Illumina NovaSeq 6000 platform, generating 150 bp paired-end reads. Raw reads were filtered and aligned to the mouse reference genome (mm10) using HISAT2. Differential expression analysis was performed using the DESeq2 R package, with a threshold of |log2FoldChange| > 1 and adjusted p-value < 0.05 for identifying significantly differentially expressed genes. Gene Ontology (GO) enrichment and Kyoto Encyclopedia of Genes and Genomes (KEGG) pathway analyses were performed using the clusterProfiler R package. Volcano plots and heatmaps were generated using the ggplot2 R package.

### Fabrication of MN arrays

Dissolvable MN arrays (10 × 10 array, 100 needles per patch) were fabricated using a polydimethylsiloxane (PDMS) mold. The mold was prepared by pouring PDMS over a master template and curing at 70 °C for 2 h. A solution containing 15% w/v PVA and 2% w/v IdE@E (20 mg/mL, equivalent to 2 mg IdE@E per array) in deionized water was prepared, thoroughly mixed, and carefully pipetted into the PDMS mold (100 μL per array). Based on the estimated mass of a single exosome, this formulation theoretically yields approximately 2 × 10^14^ total exosomes per array, resulting in a theoretical loading of 2 × 10^12^ exosomes per needle. After removing the bubbles via vacuum evaporation, the needles were dried at 25 °C for at least 24 h. This mild fabrication process (room temperature drying, aqueous environment) and the use of PVA, a well-established biocompatible polymer known for its gentle nature in biological applications, were designed to maintain the integrity and biological activity of IdE@E throughout the MN preparation. The preservation of IdE@E functionality was evidenced by the significant therapeutic effects observed in subsequent organoid and *in vivo* experiments, which would be unlikely if substantial structural damage had occurred during MN preparation.

### Characterization of MN arrays

The morphology of the MN arrays was examined using scanning electron microscopy (SEM). The distribution of DiO-labeled IdE@E in the MNs was visualized using fluorescence microscopy (Nikon). Mechanical strength was tested using a TA.XT plus Texture Analyzer (Stable Micro Systems) with force-displacement curves recorded. *In vitro* release studies were conducted by immersing the MN arrays in PBS at 37 °C and quantifying the released protein over time using a BCA protein assay kit.

### 3D bioprinting of *in vitro* dermis equivalent

An *in vitro* dermal equivalent was constructed using a 3D bioprinting approach. L929 cells and I-Col were utilized to prepare the bioink. Specifically, I-Col sponge was enzymatically digested in 0.5 M acetic acid solution containing pepsin (10 mg pepsin per 100 mg I-Col) for 24 h. The resulting I-Col pre-gel was subsequently neutralized using 10 M sodium hydroxide. L929 cells (passage number ≤ 6) were uniformly mixed into the neutralized I-Col pre-gel on ice to produce a cell-laden bioink with a final cell concentration of 2 × 10⁶ cells/mL. For biofabrication, a commercial multi-head 3D bioprinter (SUNP BIOMAKER 4; SunP Biotech) equipped with a micro-extrusion module was employed. PCL was melted at 80 °C and extruded to print a pre-designed Transwell-like structure, comprising a porous mesh (50 μm pore size) integrated into a square well (10 mm × 10 mm). The cell-laden bioink was then precisely extruded into this structure, following a predefined printing pattern, to achieve a uniform dermal layer with a thickness of 3 mm. After bioprinting, constructs were incubated at 37 °C for 30 min to facilitate thermal crosslinking and solidification of collagen fibrils. Subsequently, the constructs were immersed in cell culture medium (DMEM supplemented with 10% FBS and 1% penicillin-streptomycin). Medium was refreshed every two days, and the *in vitro* dermal models were cultured for 3 days before use in subsequent evaluations. MN-IdE@E arrays were gently pressed onto the surface of the skin organoids for 1 min. The arrays were then carefully removed, and the organoids were returned to the incubator and cultured for an additional 48 h under standard conditions.

### Animal treatment

Female C57BL/6 mice (6-8 weeks old, n = 6 per group) were anesthetized with isoflurane, and their dorsal skin was shaved and cleaned with 70% ethanol. Mice were randomly divided into three groups: MN-empty, MN-dEV, and MN-IdE@E. The MN arrays were applied to the prepared skin area with gentle pressure for 2 min. For intradermal injection comparison, an additional group received intradermal injections of IdE@E (ID-IdE@E) using a standard 30G needle.

### Analysis of lymph node and spleen T cells

On day 16 or 24 after the treatment, mice were euthanized, and lymph nodes and spleens were harvested. Single-cell suspensions were prepared by gently mashing the organs through a 70 μm cell strainer. Red blood cells were lysed using ACK lysis buffer. For surface staining, cells were incubated with fluorochrome-conjugated antibodies against CD3, CD4, and CD8 for 30 min at 4 °C. For intracellular cytokine staining, cells were stimulated with phorbol 12-myristate 13-acetate (50 ng/mL) and ionomycin (1 μg/mL) in the presence of GolgiPlug for 4 h. Cells were then fixed, permeabilized, and stained with anti-IFN-γ antibodies. Flow cytometry analysis was performed using a flow cytometer (BD FACSAria^™^III Cell Sorter), and data were analyzed using FlowJo software (FlowJo™ v10.10). Memory T cell subsets were analyzed by staining for CD44 and CD62L. B cell populations were assessed by staining for CD19 and CD138.

### Serum antibody detection

Blood was collected from the retro-orbital sinus of mice on day 16. Serum was separated by centrifugation at 3,000 g for 15 min at 4 °C. VZV gE-specific IgG antibodies were measured using an indirect ELISA. Briefly, 96-well plates were coated with VZV gE protein (2 μg/mL) overnight at 4 °C. After blocking, serial dilutions of serum samples were added and incubated for 2 h at room temperature. HRP-conjugated anti-mouse IgG was used as the secondary antibody. The reaction was developed using TMB substrate and stopped with 2N H_2_SO_4_. Absorbance was measured at 450 nm using a microplate reader.

### Cytokine analysis

Serum levels of IL-6 were measured using a commercial ELISA kit according to the manufacturer's instructions.

### Histopathological analysis

At the end of the experiment, major organs (heart, liver, spleen, lungs, and kidneys) were harvested and fixed in 4% paraformaldehyde for 24 h. The tissues were then dehydrated through a graded ethanol series, cleared in xylene, and embedded in paraffin. Sections (5 μm thick) were cut using a microtome and mounted on glass slides. The sections were deparaffinized, rehydrated, and stained with hematoxylin and eosin (H&E) following standard protocols. Stained sections were examined under a light microscope (Nikon) for any signs of tissue damage or inflammation. For skin sections, samples were collected from the treatment sites, processed as described above, and stained with H&E. Immunofluorescence staining was performed on skin cryosections using antibodies against CD11c and CD86.

### Approval of ethical committee

The experimental protocols involving animals were conducted in compliance with the laboratory animal care guidelines established by Beijing Institute of Technology (BIT), China. These procedures received approval from the Institutional Animal Care and Use Committee in BIT, under the ethical review code BIT-EC-SCXK (Beijing), 2021-0006-077. All efforts were made to minimize animal suffering and to adhere to the principles of humane animal experimentation.

### Statistical analysis

All data are presented as mean ± Standard Deviation (SD). Statistical analyses were performed using GraphPad Prism 8.0 software, and unpaired two-tailed Student's t-test was used. P values < 0.05 were considered statistically significant.

## Results

### Preparation and characterization of nano-IP@E

In this study, we first engineered *E. coli* through genetic modification, cloning and inserting the IFN-β gene sequence into the pET-22b(+) expression vector, enabling the expression of mouse IFN-β. Subsequently, these genetically engineered *E. coli* were processed into protoplasts. These protoplasts were then transformed into nano-scale particles (nano-IP) using ultrasonic treatment, during which VZV gE was fused to create antigen-carrying nano-protoplasts, referred to as nano-IP@E in this study. VZV gE was selected as the representative antigen model due to its significance as a key surface glycoprotein of the varicella-zoster virus and an ideal candidate for our antigen presentation system.

**Figure 2:**
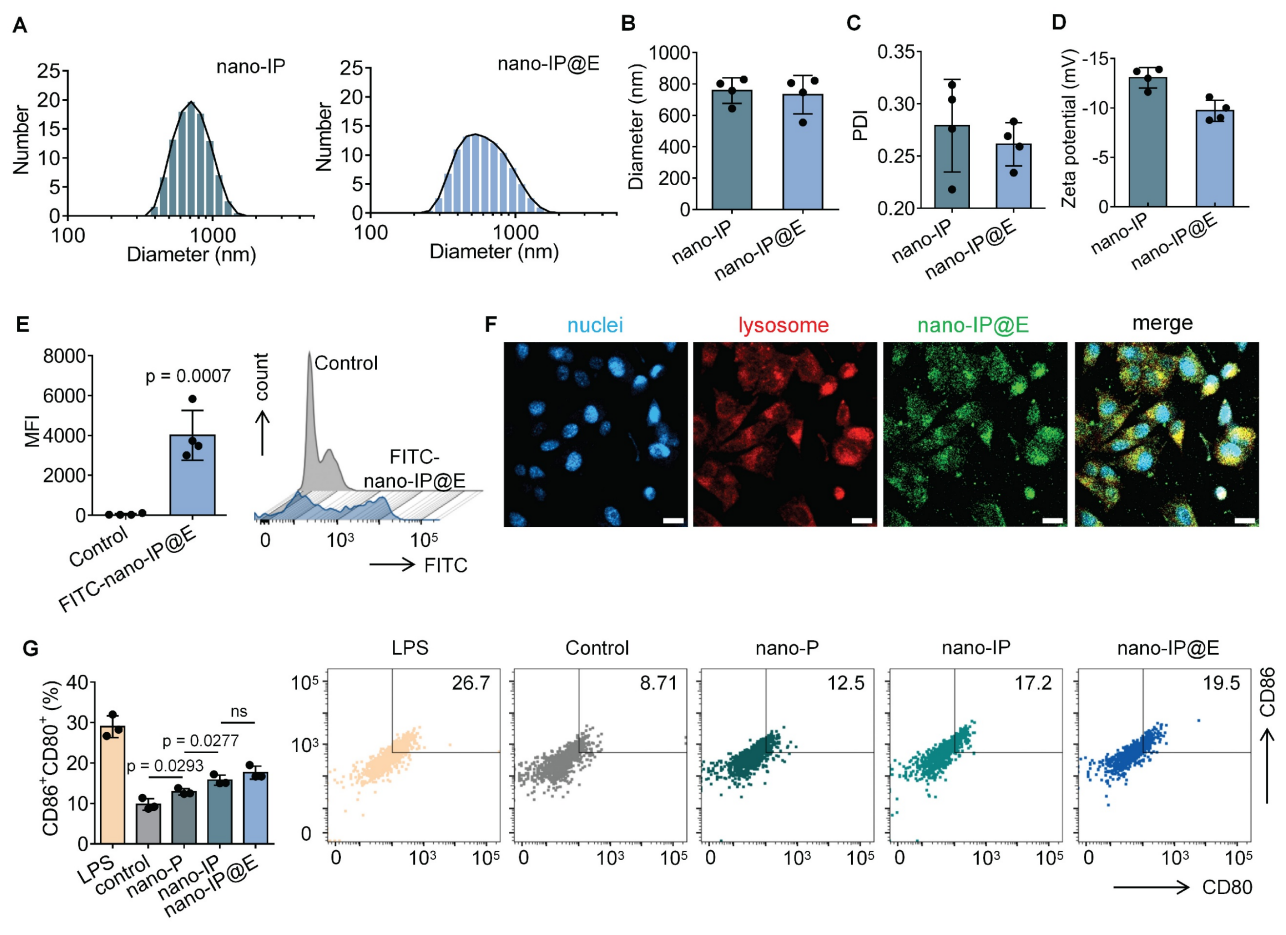
** Preparation and characterization of nano-IP@E.** (A) Particle size distribution of nano-IP and nano-IP@E measured by DLS. (B) Average diameter of nano-IP and nano-IP@E (n = 4 per group). (C) PDI values of nano-IP and nano-IP@E (n = 4 per group). (D) Zeta potential of nano-IP and nano-IP@E (n = 4 per group). (E) Flow cytometry analysis of FITC-nano-IP@E uptake by DCs, shown as MFI (n = 4 per group) and histogram. (F) Confocal microscopy images showing cellular localization of FITC-labeled nano-IP@E (green) in DCs. Cell nuclei were stained with DAPI (blue) and lysosomes were labeled in red. Scale bar = 20 μm. (G) Flow cytometry analysis of CD80^+^CD86^+^ expression on DCs after treatment with LPS (positive control), nano-P, nano-IP, or nano-IP@E, shown as percentage of double-positive cells (left) (n = 3 per group) and representative flow cytometry density plots (right). Data are presented as mean ± SD. ns: not significant.

As shown in **Figure 2A**, both nano-IP and nano-IP@E exhibited a single peak in their size distribution curves, indicating uniform particle populations. The average diameter of both formulations was approximately 700-800 nm (**Figure 2B**), with nano-IP@E showing a similar size range to nano-IP, suggesting that the fusion with VZV gE protein did not significantly alter the particle size. The PDI values for nano-IP@E was below 0.3 (**Figure 2C**), indicating relatively homogeneous size distributions. These results demonstrate that our preparation method yielded stable and uniformly sized nanoparticles, which is crucial for their potential therapeutic applications. Zeta potential measurements showed that both nano-IP and nano-IP@E maintained negative surface charges of approximately -13 mV and -10 mV, respectively (**Figure 2D**), consistent with the characteristic properties of protoplast membranes.

To validate the successful engineering of our nano-protoplasts, we conducted a comprehensive protein characterization using SDS-PAGE and ELISA analyses (**Figure S1**). The SDS-PAGE results (**Figure S1A**) revealed distinct protein profiles for nano-IP@E and nano-protoplasts without IFN-β expression (nano-P) samples. Notably, the nano-IP@E lane exhibited clear bands corresponding to the expected molecular weights of both IFN-β and VZV gE, which were absent in the nano-P control. This visual confirmation suggests the successful incorporation of both target proteins in our engineered nano-protoplasts. To quantitatively validate these observations, we performed ELISA assays for both IFN-β and VZV gE. The IFN-β ELISA results (**Figure S1B**) demonstrated significantly higher levels of IFN-β in both nano-IP and nano-IP@E samples compared to the control and nano-P groups. This finding confirms the successful expression of IFN-β in our engineered nano-protoplasts. Interestingly, there was no significant difference in IFN-β levels between nano-IP and nano-IP@E samples, suggesting that the addition of VZV gE did not interfere with IFN-β expression. The VZV gE ELISA results (**Figure S1C**) showed a significant presence of VZV gE exclusively in the nano-IP@E sample. This observation confirms the successful incorporation of the model antigen into our final nano-IP@E construct.

### Evaluation of nano-IP@E for DC stimulation

We next evaluated the ability of nano-IP@E to activate DCs. Cell viability assessments using the CCK8 assay demonstrated that DCs maintained normal viability across a range of nano-IP@E concentrations (1-20 μg/mL), with no significant cytotoxicity observed even at the highest tested concentration (**Figure S2**). Based on these results, we selected 20 μg/mL as the optimal working concentration for subsequent experiments.

To confirm cellular uptake, we labeled nano-IP@E with FITC and quantitatively analyzed uptake using flow cytometry. Results demonstrated significantly enhanced fluorescence intensity in the FITC-nano-IP@E treated cells compared to control, with mean fluorescence intensity (MFI) values of approximately 4000 for the treated group versus near-zero for controls (**Figure 2E**). To investigate the cellular fate of our engineered nano-protoplasts, we examined the uptake and intracellular localization of FITC-labeled nano-IP@E in DCs using confocal microscopy (**Figure 2F**). The images reveal widespread distribution of nano-IP@E (green) throughout the cytoplasm of DCs, with cell nuclei stained blue (DAPI) and lysosomes labeled in red. Substantial co-localization between nano-IP@E and lysosomal compartments was observed, as evidenced by the yellow signals in the merged image. This suggests that a significant portion of the nano-IP@E constructs are internalized into lysosomal compartments following cellular uptake. The presence of nano-IP@E within lysosomes could have important implications for antigen processing and presentation. However, some green fluorescence is also visible outside of the red-labeled lysosomal regions, indicating that a portion of the nano-IP@E may be present in other cellular compartments.

We then assessed the activation status of DCs following treatment with various preparations. Flow cytometry analysis of CD80 and CD86 co-expression, key markers of DC activation, revealed interesting patterns across different treatment groups (**Figure 2G**). The nano-P group showed moderate DC activation compared to the untreated control, suggesting that residual bacterial components retain some immunostimulatory capacity even after cell wall removal. More importantly, nano-IP and nano-IP@E significantly enhanced the percentage of CD80^+^CD86^+^ cells compared to both control and nano-P groups. This enhanced activation above the nano-P baseline indicates that IFN-β expression provides additional immunostimulatory effects that synergize with the inherent bacterial component-mediated activation. While LPS induced the highest level of activation as the positive control, its clinical application is limited by potential systemic inflammatory risks; in contrast, our nano-IP@E system provides a more balanced activation profile suitable for prophylactic applications while maintaining the advantage of targeted antigen and IFN-β delivery. Flow cytometry analysis showed a significant increase in the percentage of MHC-II^+^CD11c^+^ cells in the nano-IP@E treated group compared to control (**Figure S3**), further confirming the maturation and activation of DCs following nano-IP@E treatment. The above activation of DCs by nano-IP@E establishes a solid foundation for the subsequent production of IdE@E with enhanced capacity to stimulate target cells.

### Characterization and analysis of IdE@E

**Figure 3:**
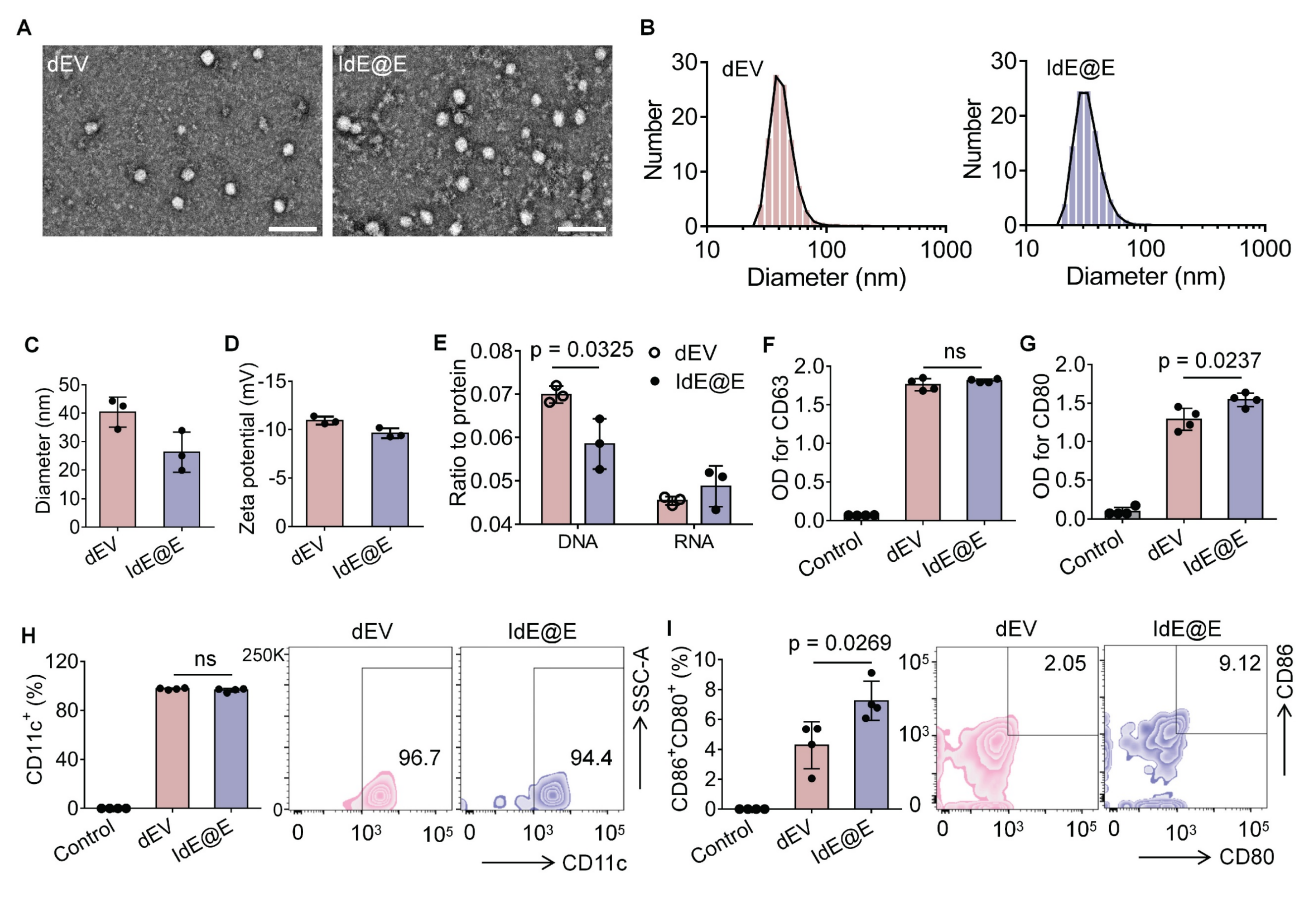
** Characterization and analysis of IdE@E.** (A) TEM images of dEV and IdE@E. Scale bar = 100 nm. (B) Particle size distribution of dEV and IdE@E. (C) Average diameter of dEV and IdE@E (n = 3 per group). (D) Zeta potential of dEV and IdE@E (n = 3 per group). (E) DNA-to-protein and RNA-to-protein ratio analysis in dEV and IdE@E (n = 3 per group). ELISA analysis of (F) CD63 and (G) CD80 expression in control (PBS), dEV, and IdE@E samples (n = 4 per group). Flow cytometry analysis of (H) CD11c^+^ and (I) CD86^+^CD80^+^ expression on dEV and IdE@E, shown as percentage of positive cells (left) (n = 4 per group) and representative density plots (right). Data are presented as mean ± SD. ns: not significant.

After extracting IdE@E from the specifically stimulated DCs, we examined their morphological and physical properties compared to exosomes secreted by unstimulated DCs (dEV). TEM images revealed that both dEV and IdE@E exhibited typical exosome morphology with spherical structures (**Figure 3A**). The particle size distribution and size characteristics of dEV and IdE@E were analyzed by DLS. As shown in the size distribution curves (**Figure 3B**), both dEV and IdE@E displayed single, narrow peaks, indicating monodisperse particle populations. The average diameter measurements (**Figure 3C**) revealed that dEV was approximately 40 nm, while IdE@E showed a slightly smaller size of around 25 nm. This size difference might reflect altered vesicle biogenesis and secretion patterns in dendritic cells upon stimulation with nano-IP@E. Both dEV and IdE@E showed comparable PDI values (approximately 0.45-0.50) with no significant difference between groups, indicating similar degrees of size distribution heterogeneity in both vesicle populations. This moderate PDI range is characteristic of naturally secreted extracellular vesicles (**Figure S4**). Both preparations exhibited characteristic negative surface charges, with zeta potentials of approximately -10 mV for dEV and -9 mV for IdE@E (**Figure 3D**). Analysis of nucleic acid content revealed a significant difference in DNA-to-protein ratio between the two preparations, with IdE@E showing a lower DNA content compared to dEV (**Figure 3E**). RNA-to-protein ratios were comparable between the two preparations, with no significant differences observed. This may indicate enhanced protein capacity and packaging into exosomes.

We next characterized the surface marker expression on both exosome types using multiple complementary techniques. ELISA analysis revealed that both dEV and IdE@E expressed comparable levels of the exosomal marker CD63, with no significant difference between the two preparations (**Figure 3F**). This finding confirms the exosomal nature of both vesicle types. Notably, IdE@E exhibited significantly higher expression of the co-stimulatory molecule CD80 compared to dEV, indicating enhanced immunostimulatory potential (**Figure 3G**). Further ELISA analyses demonstrated that both exosome types expressed similar levels of the DC marker CD11c (**Figure S5A**) and MHC-II (**Figure S5B**), with no significant differences observed between dEV and IdE@E for these crucial antigen-presenting cell markers. To validate and extend these findings, we employed Western blot analysis, which visually confirmed the increased CD80 protein levels in IdE@E compared to dEV (**Figure S6**), corroborating the ELISA results. For a more comprehensive detection of exosomal surface markers, we utilized an innovative magnetic bead-based detection method (**Figure S7**). Flow cytometry analysis using these exosome-coupled beads revealed that both dEV and IdE@E maintained high expression of CD11c, a characteristic DC marker (**Figure 3H**). This finding confirms the DC origin of both exosome types and demonstrates the preservation of this key marker throughout the engineering process. Crucially, when examining co-stimulatory molecule expression, IdE@E demonstrated significantly higher levels of CD80⁺CD86⁺ expression compared to dEV (**Figure 3I**). This result aligns with our ELISA and Western blot data, providing further evidence of the enhanced immunostimulatory potential of IdE@E. Additionally, flow cytometry analysis of MHC-II expression (**Figure S8**) revealed high levels in both dEV and IdE@E, with a slight, non-significant increase in IdE@E. Importantly, both dEV and IdE@E demonstrated significantly higher MHC-II expression compared to the control (bare beads). This preservation of MHC-II expression across both exosome types, combined with the previously observed enhanced levels of CD80/CD86 in IdE@E, strongly suggests an improved capacity for antigen presentation and T cell activation.

### DC characteristics in fibroblasts induced by IdE@E

**Figure 4:**
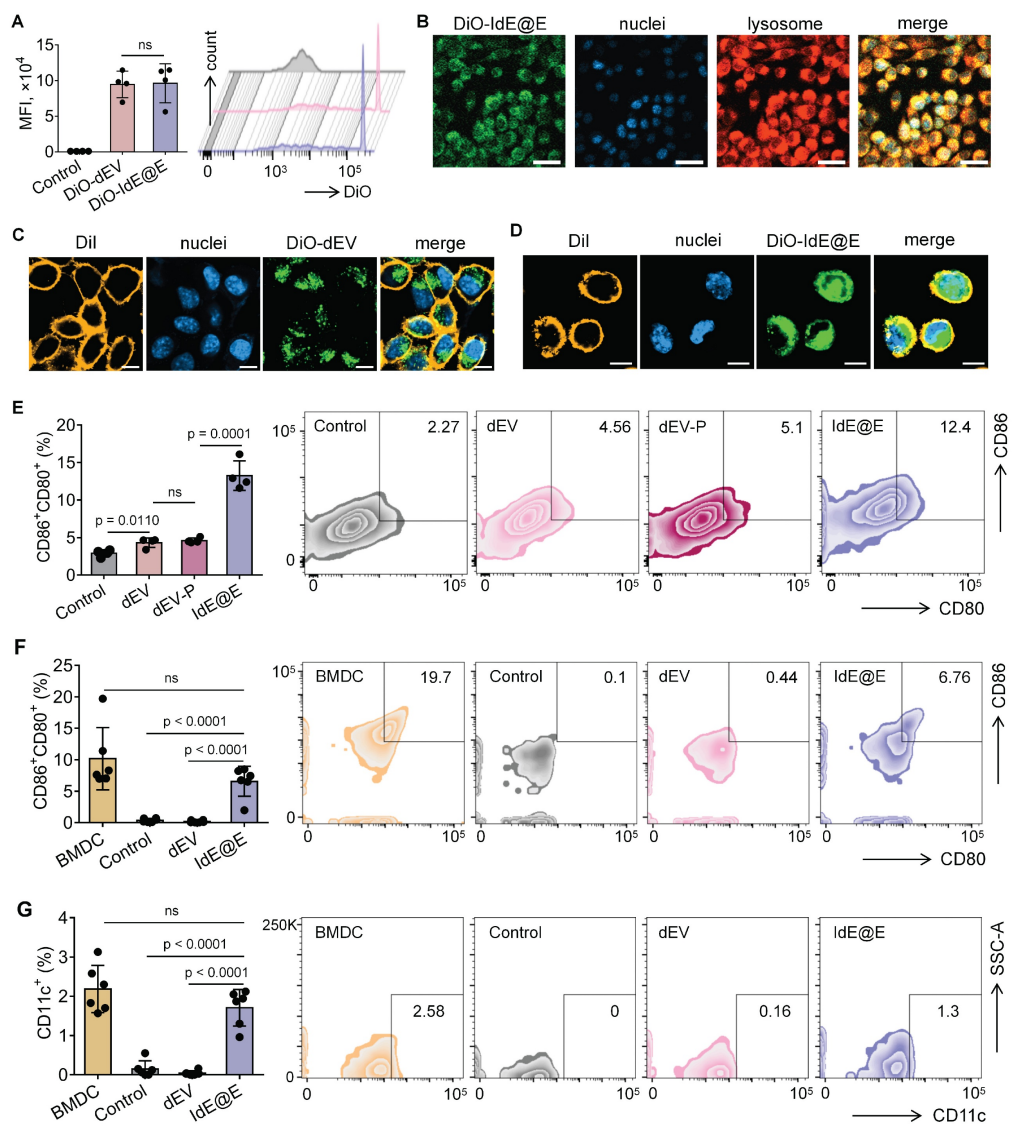
** Reprogramming of DC characteristics from fibroblasts by IdE@E.** (A) Flow cytometry analysis of DiO-labeled dEV and IdE@E uptake by L929 cells, shown as MFI (left) (n = 4 per group) and representative histograms (right). (B) Confocal microscopy images showing cellular localization of DiO-labeled IdE@E (green), nuclei (blue), and lysosomes (red), with merged image. Scale bar = 50 μm. Confocal microscopy images showing (C) DiO-dEV and (D) DiO-IdE@E uptake by cells labeled with DiI cell membrane stain (orange), nuclei (blue), and merged image. Scale bar = 10 μm. (E) Flow cytometry analysis of CD80^+^CD86^+^ expression in control, dEV, dEV-P, and IdE@E treated L929 cells, shown as percentage of double-positive cells (left) (n = 4 per group) and representative density plots (right). Comparison of (F) CD80^+^CD86^+^ and (G) CD11c^+^ expression between BMDCs and primary skin fibroblasts treated with control, dEV, or IdE@E, shown as percentage of double-positive cells (left) (n = 6 per group) and representative density plots (right). Data are presented as mean ± SD. ns: not significant.

To investigate the ability of IdE@E to induce DC-like characteristics in fibroblasts, we first evaluated the cellular uptake efficiency of our exosome preparations. We labeled both dEV and IdE@E with the fluorescent dye DiO and tracked their internalization by L929 fibroblast cells. Flow cytometry analysis revealed comparable uptake of both exosome types, with mean fluorescence intensity (MFI) values of approximately 9 × 10^4^ for both DiO-dEV and DiO-IdE@E treated cells, significantly higher than control cells (**Figure 4A**). This indicates efficient internalization of both exosome preparations by fibroblasts.

Confocal microscopy further confirmed the successful uptake of DiO-labeled IdE@E (green) by L929 cells, with fluorescent signals distributed throughout the cytoplasm and partially co-localizing with lysosomes (red), while cell nuclei were counterstained with DAPI (blue) (**Figure 4B**). Similar distribution patterns were observed for DiO-labeled dEV (**Figure S9**), confirming comparable cellular uptake mechanisms for both exosome types. Next, confocal microscopy images also revealed significant co-localization between DiO-labeled exosomes and DiI-labeled cell membranes, as evidenced by the yellow signals in the merged images. This co-localization pattern was observed in both dEV (**Figure 4C**) and IdE@E (**Figure 4D**), indicating successful membrane fusion events. Notably, green fluorescence signals were also detected within the cell boundaries, suggesting effective delivery of exosomal contents into the cytoplasm following membrane fusion. The similar distribution patterns observed for both dEV and IdE@E indicate that our engineering process does not compromise the natural fusion capability of the exosomes.

We next examined whether IdE@E could induce DC-like phenotypic changes in fibroblasts. Flow cytometry analysis of L929 cells after 48 h treatment revealed that IdE@E significantly increased the expression of the co-stimulatory molecules CD80 and CD86 compared to all other treatment groups (**Figure 4E**). Notably, while dEV treatment showed a modest but significant increase in CD80⁺CD86⁺ expression compared to control, the effect of IdE@E was substantially more pronounced, indicating its superior ability to induce DC-like phenotypic changes in fibroblasts. Interestingly, despite these significant phenotypic changes at the molecular level, bright-field microscopy revealed no substantial morphological differences between control, dEV-treated, and IdE@E-treated L929 cells (**Figure S10**). This observation suggests that IdE@E induces functional changes in fibroblasts without dramatically altering their overall cellular morphology, supporting our concept of creating "pseudo-DCs" rather than complete cellular transformation. To validate these findings in a more physiologically relevant system, we isolated primary skin fibroblasts. Flow cytometry analysis demonstrated that IdE@E treatment significantly increased CD80⁺CD86⁺ expression in primary fibroblasts compared to control and dEV treatments (**Figure 4F**). While untreated natural bone marrow-derived dendritic cells (BMDCs) showed higher baseline expression of CD80⁺CD86⁺, there was no statistically significant difference between BMDCs and IdE@E-treated fibroblasts, suggesting that IdE@E effectively induced a DC-like phenotype in these cells. Similarly, IdE@E treatment significantly increased CD11c expression in primary fibroblasts compared to control and dEV treatments (**Figure 4G**). Again, while untreated natural BMDCs showed higher baseline CD11c expression, there was no statistically significant difference between BMDCs and IdE@E-treated fibroblasts. These results demonstrate that IdE@E can effectively induce DC-like characteristics in both fibroblast cell lines and primary fibroblasts. The membrane fusion capability of IdE@E likely contributes to its enhanced efficacy in transferring immunostimulatory components to recipient cells. IdE@E treatment can transform fibroblasts into functional antigen-presenting pseudo-DCs without complete cellular reprogramming, potentially enhancing local immune responses.

**Figure 5:**
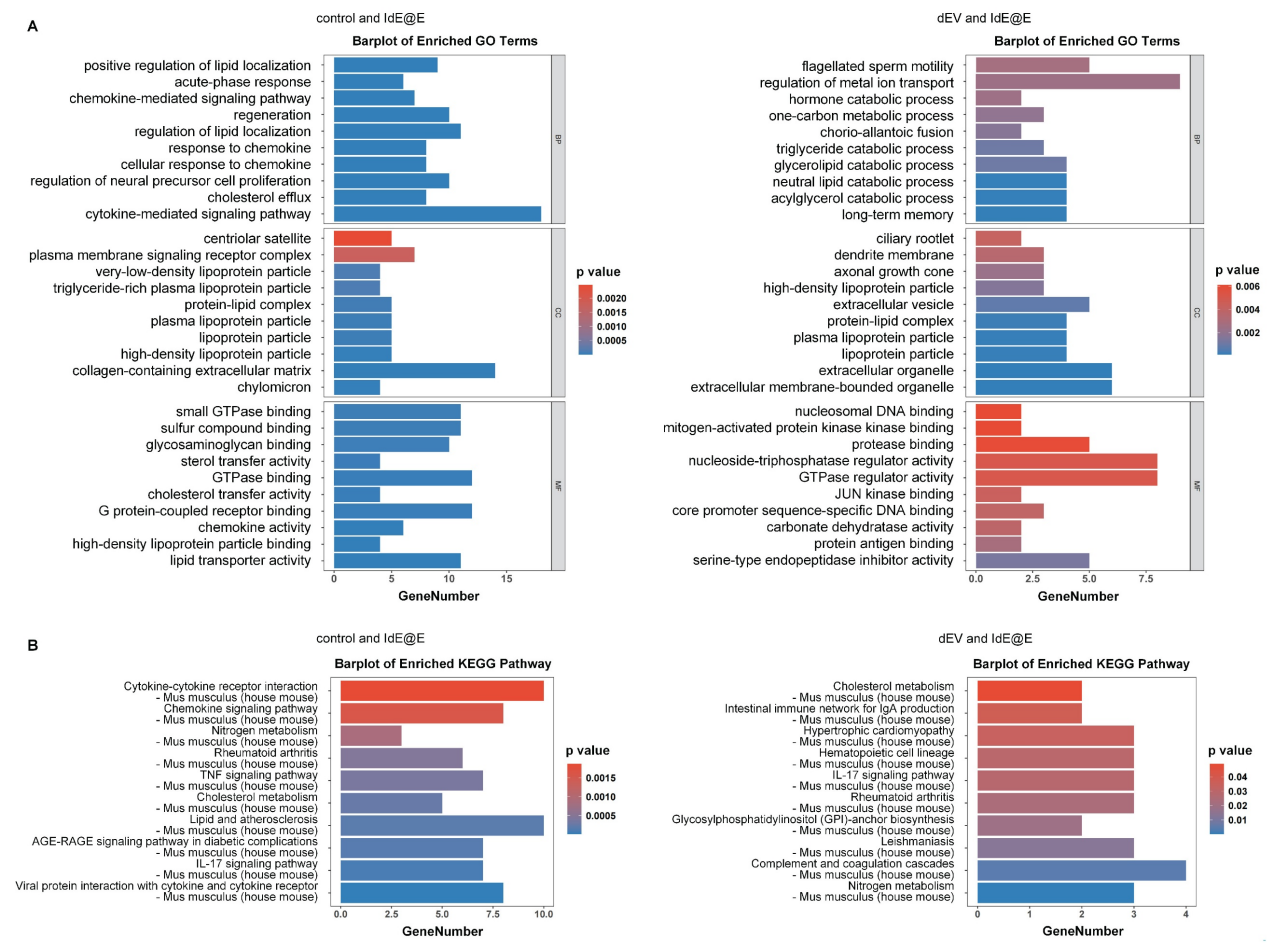
** Transcriptome analysis of IdE@E-treated fibroblasts.** (A) GO enrichment analysis showing significantly enriched biological processes, cellular components, and molecular functions in IdE@E-treated cells compared to control (left) and dEV-treated cells (right). Bar length represents gene number, and color indicates p value. (B) KEGG pathway enrichment analysis showing significantly enriched pathways in IdE@E-treated cells compared to control (left) and dEV-treated cells (right). Bar length represents gene number, and color indicates p value.

To further explore the effects of IdE@E treatment on cellular function, we conducted transcriptome sequencing of L929 cells. While our primary focus remains on the phenotypic shift towards antigen-presenting capabilities, these data provide insights into the broader cellular changes induced by IdE@E, supporting our concept of pseudo-DCs. Upon further exploration, transcriptome sequencing results revealed significant effects of IdE@E treatment on cellular gene expression of L929. The volcano plots (**Figure S11**) clearly demonstrate the differential gene expression between treatment groups. Compared to the control and dEV groups, the IdE@E-treated group exhibited more pronounced changes in gene expression, particularly evident in the direct comparison between IdE@E and dEV. Notably, key genes such as Egr1, Ccn1, H1f0, and Fos were significantly upregulated in the IdE@E-treated group, while certain genes like Gm3839 and Serpina1a showed downregulation. Then we conducted GO enrichment analysis (**Figure 5A and Figure S12**) and KEGG pathway enrichment analysis (**Figure 5B and Figure S13**) on the gene expression changes following dEV and IdE@E treatment, revealing their distinct roles in cellular function regulation. GO enrichment analysis demonstrated that, compared to the control group, dEV treatment significantly upregulated genes associated with interferon-β response, STAT protein tyrosine phosphorylation, ion transport, and cellular secretion. Additionally, it influenced genes related to nervous system development and myelination, suggesting that dEV may play crucial roles in immune regulation and nervous system function. In contrast, IdE@E treatment markedly upregulated genes involved in lipid localization, acute-phase response, chemokine signaling pathways, and cellular regeneration. It also affected genes related to neural precursor cell proliferation and cholesterol efflux. These findings indicate that IdE@E may exert broader regulatory effects on immune modulation, cellular regeneration, and lipid metabolism. Further comparison between dEV and IdE@E effects revealed that IdE@E specifically upregulated genes associated with lipid metabolism, hormone catabolic processes, and memory formation. It also influenced genes related to cellular structures (such as cilia and dendrites) and extracellular vesicles, highlighting IdE@E's unique role in metabolic regulation and neuroplasticity. KEGG pathway enrichment analysis further corroborated these observations. dEV significantly activated the JAK-STAT signaling pathway, endocannabinoid signaling pathway, and dopaminergic synapses, indicating its role in immune regulation and neurotransmitter signal transduction. IdE@E, on the other hand, significantly activated cytokine-cytokine receptor interactions, chemokine signaling pathways, and the TNF signaling pathway, while also influencing metabolic pathways such as nitrogen metabolism and cholesterol metabolism. This suggests that IdE@E may have more extensive effects on immune regulation and metabolic control. Notably, compared to dEV, IdE@E specifically activated cholesterol metabolism, the intestinal immune network for IgA production, and the IL-17 signaling pathway, further confirming its unique role in metabolic regulation and immune modulation. These results reveal that while both dEV and IdE@E possess immunomodulatory properties, IdE@E exhibits more extensive and distinctive effects on metabolic regulation, cellular regeneration, and activation of specific immune pathways. These findings provide an important molecular basis for the potential application of IdE@E in immunotherapy and metabolic disease treatment, while also guiding future research into the mechanisms of IdE@E action. In addition, The heatmaps (**Figure S14**) further corroborate these observations. The IdE@E-treated group presents a distinct gene expression pattern compared to both control and dEV groups, characterized by clear clusters of up- and down-regulated genes. This differentiated expression profile emphasizes the unique role of IdE@E in inducing cellular phenotype transformation. These transcriptomic data provide other in-depth insights into the IdE@E-induced cellular phenotype shift. The marked upregulation of Egr1 (Early Growth Response 1) is particularly noteworthy, as this gene is involved in cellular differentiation and immune response. Its increased expression supports the hypothesis that IdE@E promotes a transition towards a more immunogenic phenotype. Moreover, the upregulation of genes such as Ccn1 and Fos, which are involved in cell proliferation and differentiation, further substantiates IdE@E's capacity to induce cells into a more active, potentially more immunogenic state. The significant differences between IdE@E and dEV treatment effects indicate that these engineered exosomes possess unique properties that surpass those of standard DC-derived exosomes. The extensive changes in gene expression suggest that IdE@E treatment may induce multifaceted functional alterations in cells, potentially including enhanced antigen presentation capabilities, increased co-stimulatory molecule expression, and altered cytokine production. Collectively, these transcriptome analysis results provide robust molecular evidence for the ability of IdE@E to induce cells towards a more immunogenic phenotype. This finding has important implications for enhancing local immune responses in tissues and may play a crucial role in various therapeutic contexts, providing a significant basis for developing novel immunomodulatory strategies. Transcriptome analysis revealed that IdE@E treatment significantly upregulated genes associated with immune response, cell differentiation, and metabolic regulation, including Egr1, Ccn1, and Fos. While these transcriptomic changes provide valuable insights into the cellular responses to IdE@E treatment, it's important to note that they represent a partial functional adaptation rather than complete cellular reprogramming. This aligns with our concept of inducing 'pseudo-DCs' - fibroblasts that acquire key antigen-presenting capabilities without fully transforming into professional APCs.

While classical markers such as CD40 were not among the most significantly upregulated genes, IdE@E treatment induced changes in several gene networks functionally related to antigen presentation. Additionally, our pathway analysis revealed significant enrichment of chemokine signaling and JAK-STAT pathways, which are critical for DC activation and immune cell recruitment. These findings support our concept of "pseudo-DCs" - cells that acquire specific functional aspects of DCs while maintaining their original identity. This selective adoption of DC-like features is consistent with our functional observations that IdE@E-treated fibroblasts can effectively present antigens and stimulate T cell responses. The transcriptome data thus provide molecular evidence for a functional adaptation that enhances immunostimulatory capacity without requiring complete cellular transdifferentiation.

Although IdE@E has been shown to significantly induce DC-like characteristics in fibroblasts, we were interested in determining whether these exosomes would further enhance the activation state of the parent dendritic cells themselves. Flow cytometry analysis of CD80⁺CD86⁺ expression in dendritic cells treated with either dEV or IdE@E showed no significant differences between the treatment groups (**Figure S15**). This finding suggests that while IdE@E can effectively induce DC-like phenotypic changes in fibroblasts, they do not further enhance the expression of co-stimulatory molecules in dendritic cells themselves.

### Characterization of MN-IdE@E

**Figure 6:**
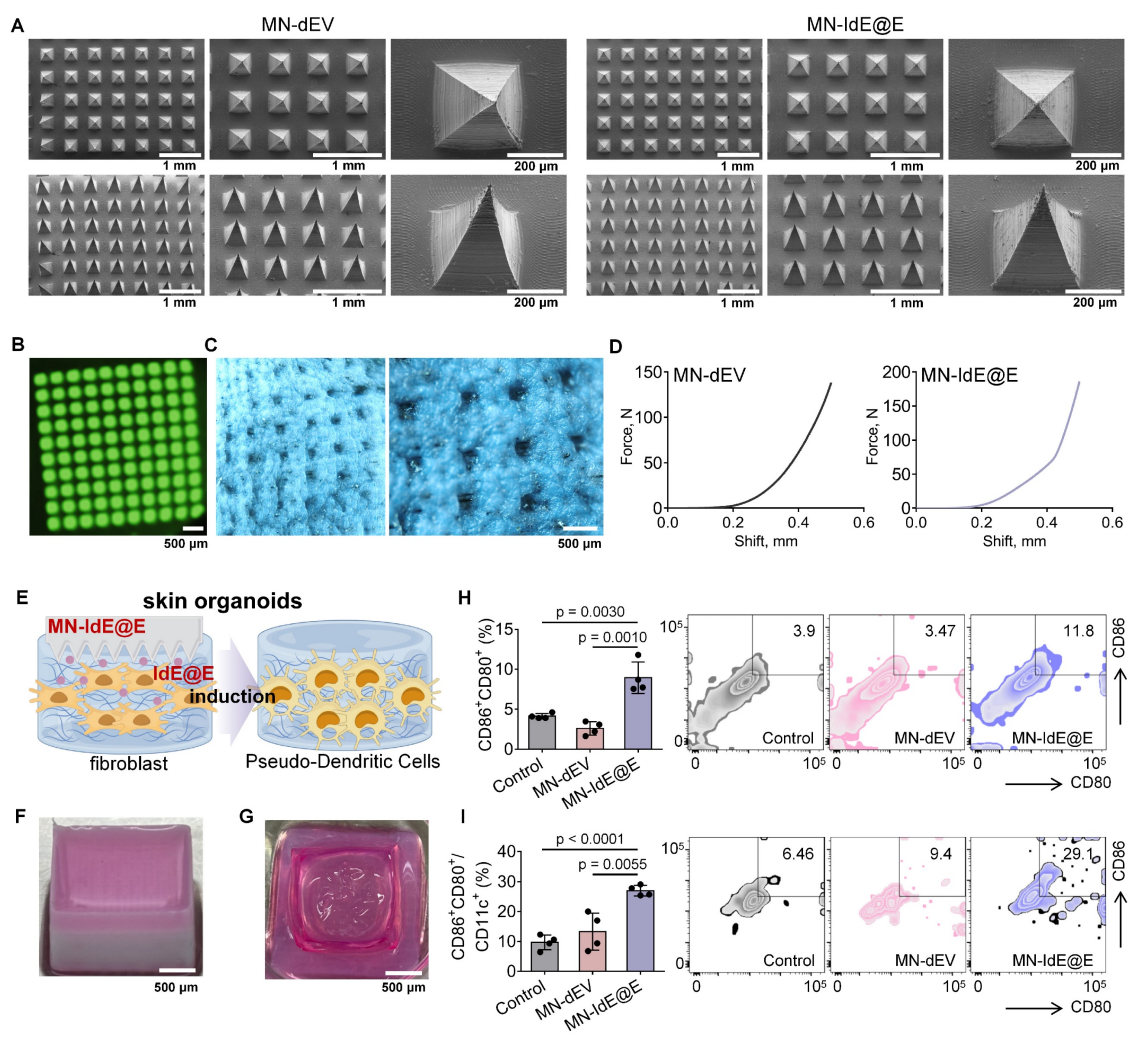
** Characterization of MN-IdE@E and efficacy in skin organoid model.** (A) SEM images of MN-IdE@E. (B) Top-view fluorescence microscopy image of DiO-labeled IdE@E distribution in MNs under UV illumination. (C) Optical microscopy images of mouse skin after MN-IdE@E application. (D) Force-displacement curves of MN-dEV and MN-IdE@E. (E) Schematic of MN-IdE@E application to skin organoid model, which was created using Figdraw. (F) Photograph of 3D-printed skin organoid. (G) Photograph of dissolved MN-IdE@E after insertion into skin organoid. (H) Flow cytometry analysis of CD80^+^CD86^+^ cells in skin organoid after MN-IdE@E treatment (n = 4 per group). (I) Flow cytometry analysis of CD80^+^CD86^+^CD11c^+^ cells in skin organoid after MN-IdE@E treatment (n = 4 per group). Data are presented as mean ± SD.

SEM images revealed that the two types of dissolvable MNs loaded with dEV and IdE@E (MN-dEV and MN-IdE@E) were morphologically very similar, both exhibiting clear, intact, and uniform pyramid array structures (**Figure 6A and Figure S16**). This observation indicates that the incorporation of exosomes did not significantly alter the physical structure of the MNs, thus preserving their fundamental function as a transdermal delivery system. This structural integrity is crucial for ensuring consistent and effective penetration of the skin barrier. Under UV illumination, the top-view fluorescence image of dissolvable MNs loaded with DiO-labeled IdE@E displayed uniformly distributed green fluorescent spots (**Figure 6B**), representing the distribution of fluorescently labeled exosomes within the MNs. Side-view fluorescence microscopy of DiO-labeled IdE@E-loaded MNs further confirmed the uniform distribution of exosomes throughout the MN structure (**Figure S17**), supporting the potential for efficient and uniform transdermal delivery. Optical microscopy was used to observe the condition of MNs after insertion into mouse skin (**Figure 6C**). The clear and deep micropores visible in the images preliminarily indicate that MN-IdE@E can successfully penetrate the skin surface, possessing sufficient mechanical strength. This observation is critical for ensuring that the MNs can effectively deliver their payload to the target skin layers. Furthermore, two force-displacement curves from MN-dEV and MN-IdE@E reaffirm their adequate mechanical strength and penetration capability (**Figure 6D**). The sharp rise in the curves at 0.3-0.4 mm displacement suggests an effective penetration depth of 0.5-0.6 mm, which is ideal for delivering IdE@E to the epidermis and dermis where immune cells are concentrated. Analysis of the mechanical properties showed that when the total array force was distributed across the needle array (100 needles), the apparent force per needle reached approximately 1.4 N and 1.8 N for MN-dEV and MN-IdE@E respectively. These values satisfy the reported threshold force required for skin penetration. The comparable force-displacement profiles between the two formulations indicates that the addition of IdE@E maintained the essential mechanical properties of the microneedles, ensuring reliable skin penetration capability. According to **Figure S18**, the IdE@E-loaded soluble MNs exhibit a distinctive biphasic release profile: approximately 40% of the drug is rapidly released within the first 5 h, achieving immediate immune activation, followed by a decelerated release rate with cumulative release reaching 75% at 24 h and near-complete release (95%) by 48 h. This pattern is particularly suitable for immunotherapy applications, the initial burst release triggers immune responses while the subsequent sustained release maintains immune stimulation. Although the physical release process is completed within 48 h, its biological effects can persist for an extended duration. This characteristic provides an ideal foundation for prophylactic applications, where a single administration can establish long-lasting protective immunity.

### Efficacy of MN-IdE@E in a skin organoid model

We then established skin organoids using 3D printing technology. **Figure 6E** schematically illustrates the process of MN-IdE@E inducing internal fibroblasts into antigen-presenting pseudo-DCs in the skin organoid model. This model provides a more physiologically relevant platform for studying the effects of IdE@E delivery. **Figure 6F** shows a photograph of our skin organoid, while **Figure 6G** displays a photograph of MN-IdE@E fully dissolved after insertion into the skin organoid, collectively providing a research platform that more closely mimics the *in vivo* environment for subsequent experiments. This series of optical microscopy images (**Figure S19**) illustrates that fibroblasts within skin organoid model preserve a normal condition and a high cellular density across different treatment modalities. Flow cytometry results demonstrate the proportions of CD80^+^CD86^+^ (**Figure 6H**) and CD80^+^CD86^+^CD11c^+^ (**Figure 6I**) cells in different treatment groups within the skin organoid model. The results show that the MN-IdE@E group had significantly higher proportions of these two cell types compared to other groups. This finding is consistent with previous *in vitro* experimental results, further confirming that IdE@E can effectively induce fibroblasts to acquire a DC-like phenotype, even in a more complex three-dimensional skin organoid model. The increased expression of these surface markers suggests a shift towards a more immunogenic phenotype, which could have important implications for enhancing local immune responses in the skin.

### *In situ* reprogramming effect of MN-IdE@E on murine skin fibroblasts

**Figure 7:**
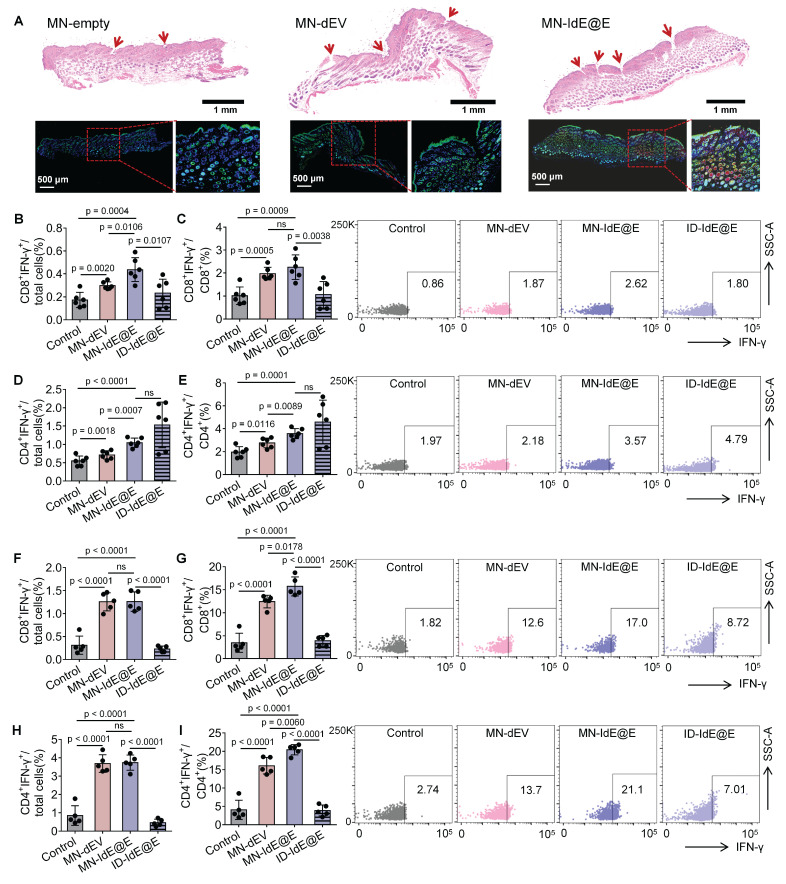
***In vivo* evaluation of MN-delivered IdE@E for enhancing adaptive immunity.** (A) H&E staining (top) and immunofluorescence images (bottom) of skin sections after treatment with empty MNs, MN-dEV, or MN-IdE@E. Red arrows indicate MN penetration sites. (B-C) Percentage of CD8^+^ IFN-γ^+^ cells among total cells (B) and CD8^+^ T cells (C) in lymph nodes 16 days post-immunization, with representative flow cytometry plots (right) for control, MN-dEV, MN-IdE@E, and ID-IdE@E groups (n = 6 per group). (D-E) Percentage of CD4^+^ IFN-γ^+^ cells among total cells (D) and CD4^+^ T cells (E) in lymph nodes 16 days post-immunization, with representative flow cytometry plots (right) for all groups (n = 6 per group). (F-G) Percentage of CD8^+^ IFN-γ^+^ cells among total cells (F) and CD8^+^ T cells (G) in spleens 16 days post-immunization, with representative flow cytometry plots (right) for all groups (n = 5 per group). (H-I) Percentage of CD4^+^ IFN-γ^+^ cells among total cells (H) and CD4^+^ T cells (I) in spleens 16 days post-immunization, with representative flow cytometry plots (right) for all groups (n = 5 per group). For spleen analysis in this stage, one mouse was used for organ safety evaluation, resulting in n = 5 for splenic T cell analysis. Data are presented as mean ± SD. ns: not significant.

We further evaluated the efficacy of MN-IdE@E in inducing intradermal fibroblasts in murine skin (**Figure 7A**). The experimental protocol involved three distinct treatments: empty MNs (MN-empty), MN-dEV, and MN-IdE@E. H&E staining was employed to visualize the application sites of the MNs, with the red arrows indicating the presence of evident puncture tracks, signifying effective penetration. Immunofluorescence staining was utilized to assess cellular markers, where blue fluorescence denotes nuclear staining (DAPI), green fluorescence represents the DC marker CD11c, and red fluorescence indicates the activation marker CD86. Comparative analysis of the three treatment modalities revealed that the MN-empty group exhibited minimal expression of CD11c and CD86. The MN-dEV group demonstrated a slight increment in marker expression, whereas the MN-IdE@E group exhibited the most substantial increase, particularly evident in the magnified insert. These findings conclusively demonstrate that MN-IdE@E is the most potent in inducing the expression of DC and activation markers in skin cells, presumably fibroblasts. This observation corroborates our hypothesis that IdE@E administered via soluble MNs can effectively transpose skin fibroblasts into a DC-like phenotype with enhanced immunogenicity, referred to as pseudo-DCs, *in vivo*.

### *In vivo* immune responses

We analyzed the lymphocytes in lymph nodes and spleens using flow cytometry, with the overall gating strategy depicted in **Figure S20**. **Figure S21** demonstrates the effects of various treatments on T cell subsets in lymph nodes of C57 mice 16 days post-immunization. For CD8^+^ T cells, MN-IdE@E treatment significantly increased their proportion compared to control and MN-dEV groups. Importantly, MN-IdE@E also showed a significant increase in CD8^+^ T cells compared to ID-IdE@E, indicating that MN delivery is more effective than conventional intradermal injection in enhancing CD8^+^ T cell proportions. This difference likely reflects the superior intradermal delivery efficiency of MNs compared to conventional intradermal injection using syringes. Regarding CD4^+^ T cells, there were no significant differences between control, MN-dEV, and ID-IdE@E groups. However, the MN-IdE@E group showed a slight but significant decrease in CD4^+^ T cell proportion compared to control and MN-dEV.

We analyzed IFN-γ production by CD8⁺ and CD4⁺ T cells in lymph nodes and spleens at 16 and 24 days post-immunization to evaluate the dynamics of T cell responses induced by MN-IdE@E treatment. In lymph nodes at 16 days, MN-IdE@E group showed significantly higher percentages of CD8⁺IFN-γ⁺ cells among total cells compared to the control group (**Figure 7B**). This trend persisted in the CD8⁺ T cell population, with MN-IdE@E group showing significant increases compared to the control (**Figure 7C**). Notably, the MN-IdE@E group consistently outperformed the ID-IdE@E group. In spleens at 16 days, both MN-IdE@E and MN-dEV groups exhibited significantly higher percentages of CD8⁺IFN-γ⁺ cells compared to the control and ID-IdE@E groups (**Figure 7F-G**). The enhanced immune responses observed with MN delivery can be attributed to several key advantages over traditional intradermal injection. Our MN array enables precise and consistent delivery to immune-cell-rich skin layers, overcoming the technical challenges and operator dependency of traditional injection. The dissolving MN system ensures minimal loss of engineered exosomes, whereas traditional injection often suffers from significant backflow and leakage. Moreover, the array design with 100 needles per patch creates multiple penetration points, enabling wider and more even distribution of IdE@E throughout the target tissue, thereby enhancing interaction between exosomes and immune cells. Comparing the 16-day and 24-day time points reveals interesting dynamics. In lymph nodes, the robust CD8⁺ T cell response seen at 16 days for MN-IdE@E was maintained at 24 days (**Figure S22A-B**), indicating a sustained effect. Similarly, in spleens, the strong CD8⁺ T cell response observed at 16 days for MN-IdE@E and MN-dEV groups persisted at 24 days (**Figure S23A-B**), suggesting durable systemic immunity. CD4⁺ T cell responses showed different temporal patterns. In lymph nodes, the CD4⁺ T cell activation seen at 16 days for MN-IdE@E was not as pronounced at 24 days (**Figure 7D-E and Figure S22C-D**), indicating a potential shift in the immune response over time. In spleens, the robust CD4⁺ T cell response observed at 16 days for MN-IdE@E and MN-dEV groups showed some decline by 24 days (**Figure 7H-I and Figure S23C-D**), but remained elevated compared to the control. These dynamics suggest that MN-IdE@E induces a more balanced and sustained T cell response in both lymphoid and peripheral tissues. The persistence of elevated CD8⁺ T cell responses, particularly in the spleen, indicates the potential for long-lasting systemic immunity. The evolving nature of CD4⁺ T cell responses over time may reflect a shift from initial activation to memory formation or regulatory functions.

To further elucidate the specific effects of our IdE@E formulation and the MN delivery system, we conducted an additional experiment 16 days post-immunization, introducing a supplementary control group (dEV-P) alongside repeated the control and IdE@E groups (**Figure S24**). CD8⁺ T cell responses showed a consistent gradual increase across the groups (**Figure S24A-B**). The percentage of CD8⁺IFN-γ⁺ cells among total cells was significantly higher in the IdE@E group compared to both the control and dEV-P groups. Similarly, when analyzing CD8⁺IFN-γ⁺ cells as a percentage of CD8⁺ T cells, both dEV-P and IdE@E groups showed significant increases compared to the Control. CD4⁺ T cell responses followed a similar pattern (**Figure S24C-D**).

We analyzed the formation of memory T cell subsets in both lymph nodes and spleens 24 days post-immunization to assess the long-term impact of MN-IdE@E treatment (**Figure S25 and S26**). In lymph nodes, the percentage of CD44⁺CD62L⁻ effector memory T cells among CD3⁺ T cells was significantly increased in both MN-dEV and MN-IdE@E groups compared to the control. The MN-dEV and MN-IdE@E groups showed similar levels of effector memory T cells, both higher than the control group. Interestingly, no significant differences were observed in the percentages of CD44⁺CD62L⁺ central memory T cells among the three groups in lymph nodes. In contrast, analysis of splenic memory T cells revealed a more pronounced effect of the MN-IdE@E treatment. The percentage of CD44⁺CD62L⁻ effector memory T cells in the spleen was significantly higher in the MN-IdE@E group compared to both the MN-dEV and control groups. The MN-dEV group also showed a significant increase compared to the control. Similar to the lymph nodes, no significant differences were observed in the percentages of CD44⁺CD62L⁺ central memory T cells among the three groups in the spleen. Notably, the MN-IdE@E group exhibited a markedly larger effector memory population in the spleen compared to MN-dEV and control groups. The increased presence of effector memory T cells, particularly in the spleen, suggests a robust and sustained systemic immune response. This may provide rapid and effective protection against subsequent antigen encounters. The similar efficacy of MN-dEV and MN-IdE@E in generating effector memory T cells in lymph nodes, coupled with the superior effect of MN-IdE@E in the spleen, implies that the MN delivery system combined with the IdE@E formulation synergistically promotes systemic memory T cell formation.

Analysis of B cell populations in lymph nodes 24 days post-immunization revealed no significant differences in CD19⁺ B cells among the control, MN-dEV, and MN-IdE@E groups (**Figure S27A**). The percentages of CD19⁺ cells among CD3⁻ cells remained consistent across all groups. Similarly, examination of CD138⁺ cells, typically associated with plasma cells or plasmablasts, showed no statistically significant differences among the three groups (**Figure S27B**). These results indicate that at the observed time point, the treatments did not significantly alter B cell populations in the lymph nodes. This phenomenon may reflect the dynamic nature of the immune response. By 24 days post-immunization, the initial phases of B cell expansion and contraction may have concluded, with cell numbers possibly returning to homeostatic levels. In contrast, the results from the spleen showed significant differences among the control, MN-dEV, and MN-IdE@E groups. The percentage of CD19⁺ cells among CD3⁻ cells demonstrated a significant increasing trend from the control group to the MN-dEV group and further to the MN-IdE@E group (**Figure S28A**). Analysis of CD138⁺ cells (typically associated with plasma cells or plasmablasts) also revealed significant differences between groups; however, the MN-IdE@E group exhibited a downward trend (**Figure S28B**). It is noteworthy that the stability of B cell populations does not necessarily imply that the treatments had no effect on the overall humoral immune response. Some activated B cells may have differentiated into memory B cells rather than plasma cells, a change that would not be captured by the CD138 marker.

Regarding the humoral immune response, due to the loading of VZV gE-specific antigen, the MN-IdE@E treatment group demonstrated a significant advantage in inducing VZV gE-specific antibody production (**Figure S29**). Compared to the control group and dEV treatment group, which had nearly negligible OD values, the IdE@E group had significantly elevated OD values, with an average around 0.7, and individual data points were concentrated between 0.6 and 0.8.

The results in **Figure S30** suggest that neither dEV nor IdE@E treatments significantly altered the production level of IL-6. As an important inflammatory factor and immunomodulatory cytokine, the relatively stable level of IL-6 may imply that these treatments did not cause significant inflammatory responses or overactivation of the immune system.

For *in vivo* safety assessment, the weight growth curves of the mice in the three groups were very similar (**Figure S31**). This suggests that neither MN-dEV nor MN-IdE@E treatments had a significant impact on the normal growth and development of the mice. MN-dEV and MN-IdE@E treatments did not produce significant negative effects on the overall health and growth of the mice, which is of great importance for assessing the safety of these treatments. Additionally, **Figure S32** shows the HE-stained sections of major organs from mice treated with MN-IdE@E, including the heart, liver, spleen, lungs, and kidneys. Overall, the tissue structure of all organs appeared normal, with no obvious pathological changes or signs of damage.

## Discussion

The fusion of protoplasts with antigens to create nano-stimulants offers several compelling advantages over direct protein administration approaches. While free IFN-β and antigens are subject to rapid degradation and clearance in physiological conditions, our protoplast-based delivery system acts as a protective carrier that shields these bioactive components. The nanoscale size enhances cellular uptake through DCs' natural phagocytic capacity 41, 42, while the bacterial-derived nature of the platform provides additional immunostimulatory properties that synergize with IFN-β signaling. This system creates a unique "depot" effect, allowing for sustained release within cells and better mimicking physiological immune activation compared to bolus protein administration. Moreover, it mimics pathogen structures, potentially inducing a more natural immune response 19, 43.

The incorporation of IFN-β plays a crucial role in promoting DC maturation and enhancing antigen presentation capabilities 44. It is important to note that in our nano-IP@E system, IFN-β serves as an internal stimulatory signal delivered to DCs through engineered protoplasts, rather than being intended for DC secretion. Upon protoplast internalization, this delivered IFN-β enhances DC activation from within the cells, as evidenced by the increased expression of activation markers such as CD80/CD86 compared to nano-P treatment. IFN-β upregulates co-stimulatory molecules, increases MHC expression, and enhances the overall immunostimulatory capacity of DCs. This strategic delivery and activation mechanism, combined with nano-stimulants, creates a synergistic effect potentially leading to more robust and targeted immune responses.

The present study introduces a novel approach for the *in situ* reprogramming of antigen-presenting pseudo-DCs using an exosome-loaded MN array system. This method addresses several limitations associated with conventional strategies that rely on *ex vivo* differentiation and reinfusion of DCs. Our findings demonstrate that engineered exosomes, derived from DCs stimulated by nano-protoplasts expressing IFN-β and loading VZV gE, can effectively induce fibroblasts to adopt a DC-like phenotype. This reprogramming process occurs without complete cellular transdifferentiation, suggesting that a partial phenotypic shift may suffice for functional immune activation. The ability to induce antigen-presenting pseudo-DCs *in situ* offers a promising avenue for enhancing local immune responses, potentially revolutionizing immunotherapy by utilizing endogenous cellular resources. This approach not only has potential therapeutic applications but also offers significant promise for preventive strategies in healthy individuals, potentially enhancing natural defense mechanisms against various conditions before they manifest.

The choice of skin as the target site for this reprogramming strategy is supported by its rich fibroblast content and accessibility for non-invasive interventions 45. Furthermore, the membrane fusion capabilities of exosomes with target cells in the skin microenvironment potentially enhance the efficiency of cellular phenotype modification, contributing to the overall effectiveness of this preventive approach. Our study successfully demonstrated the integration of IdE@E into dissolvable MNs, facilitating efficient transdermal delivery and subsequent reprogramming of DC-like phenotypes from fibroblasts within both skin organoid models and murine skin. The MN system ensured consistent penetration and release of exosomes, as evidenced by the observed immune activation in treated tissues. This approach not only circumvents the logistical challenges of *ex vivo* cell manipulation but also provides a scalable framework adaptable to diverse antigen models, making it a versatile tool for precision immunotherapy.

The comprehensive characterization of IdE@E, including their enhanced expression of co-stimulatory molecules and antigen-specific components, underscores their potential as potent immunotherapeutic agents. The transcriptomic analysis further revealed significant gene expression changes in fibroblasts treated with IdE@E, highlighting the unique capacity of these engineered exosomes to induce a more immunogenic phenotype. The upregulation of genes involved in immune response and cellular differentiation supports the hypothesis that IdE@E promotes a transition towards a functional antigen-presenting cell state. These findings provide a robust molecular basis for the observed phenotypic shifts and suggest that IdE@E could enhance local immune responses by converting stromal cells into functional antigen-presenting cells.

The *in vivo* efficacy of MN-IdE@E was demonstrated through increased activation of T cell subsets and enhanced cytokine production in both local immune organs. The ability of MN-IdE@E to induce specific immune responses, such as increased IFN-γ production by T cells, suggests its potential application in enhancing the body's natural defense mechanisms against various potential threats, including neoplastic and infectious diseases. Importantly, the safety profile of MN-IdE@E, as indicated by stable IL-6 levels and normal tissue histology, supports its potential for clinical translation, particularly in preventive applications for healthy individuals.

## Supplementary Material

Supplemental information (Figures S1-S32) includes 32 figures providing additional experimental details, data analysis, and results. These figures encompass protein characterization, cell viability assays, microscopy images, flow cytometry analyses, gene expression analyses, MN characterization, *in vivo* immune response data, and safety evaluations. Specifically, they detail the cellular uptake and phenotype changes, transcriptomic analyses, MN array design and performance, T cell activation and memory formation in lymphoid organs, B cell responses, antibody production, cytokine levels, and histological examinations of major organs. These supplementary data support and extend the main findings presented in the paper.

## Abbreviations

BMDCs: bone marrow-derived dendritic cells
CCK-8: Cell Counting Kit-8
DAPI: 4′,6-diamidino-2-phenylindole
DC: dendritic cell
dEV: exosomes secreted by ordinary DCs
dEV-P: exosomes secreted by DCs stimulated with nano-P
DiO: 3,3'-dioctadecyloxacarbocyanine perchlorate
DiI: 1,1'-dioctadecyl-3,3,3',3'-tetramethylindocarbocyanine perchlorate
DMEM: Dulbecco's Modified Eagle Medium
EDTA: ethylenediaminetetraacetic acid
FBS: fetal bovine serum
FITC: fluorescein isothiocyanate
GO: Gene Ontology
H&E: hematoxylin and eosin
I-Col: type-I collagen
IdE@E: immunostimulatory exosomes derived from DCs stimulated by nano-IP@E
IPTG: isopropyl β-D-1-thiogalactopyranoside
KEGG: Kyoto Encyclopedia of Genes and Genomes
MHC: major histocompatibility complex
MN: microneedle
nano-IP: nano-protoplasts expressing IFN-β
nano-IP@E: nano-protoplasts expressing IFN-β and loaded with VZV gE
nano-P: nano-protoplasts without IFN-β expression
PBS: phosphate-buffered saline
PDMS: polydimethylsiloxane
PDI: polydispersity index
PVA: polyvinyl alcohol
RPMI: Roswell Park Memorial Institute
SEM: scanning electron microscopy
TEM: transmission electron microscopy
VZV gE: varicella-zoster virus glycoprotein E
