# Supplementary material for: In situ reprogramming of fibroblasts into antigen-presenting pseudo-dendritic cells via IFN-β-engineered protoplast-derived exosomes delivered by microneedle arrays to enhance adaptive immunity
Source: Theranostics. 2025 Aug 16;15(17):9179–99. doi: 10.7150/thno.115080 (PMC12439482; doi:10.7150/thno.115080)
Supplement: Supplementary file 1 — Supplemental information (Figures S1-S32) includes 32 figures providing additional experimental details, data analysis, and results. These figures encompass protein characterization, cell viability assays, microscopy images, flow cytometry analyses, gene expression analyses, MN characterization, in vivo immune response data, and safety evaluations. Specifically, they detail the cellular uptake and phenotype changes, transcriptomic analyses, MN array design and performance, T cell activation and memory formation in lymphoid organs, B cell responses, antibody production, cytokine levels, and histological examinations of major organs. These supplementary data support and extend the main findings presented in the paper. [file thnov15p9179s1.pdf]

Supplementary material for *Theranostics*

In situ **reprogramming of fibroblasts into antigen-presenting pseudo-dendritic cells via IFN-β-engineered protoplast-derived exosomes delivered by microneedle arrays to enhance adaptive immunity**

Yue Yin[a, b, †, *], Shijie Zhao[b, †], Wei Li[b], Yuan Cui[b], Thanh Loc Nguyen[c], Ge Gao[b, *]

[a] Advanced Technology Research Institute, Beijing Institute of Technology, Jinan, 250307, China.

[b] School of Medical Technology, Beijing Institute of Technology, Beijing, 100081, China.

[c] South Australian immunoGENomics Cancer Institute (SAiGENCI), Faculty of Health and Medical Sciences, The University of Adelaide, Adelaide, South Australia 5005, Australia

* Correspondence: yinyue@bit.edu.cn (Yue Yin), gaoge@bit.edu.cn (Ge Gao)

† They contributed equally to this work as co-first authors.

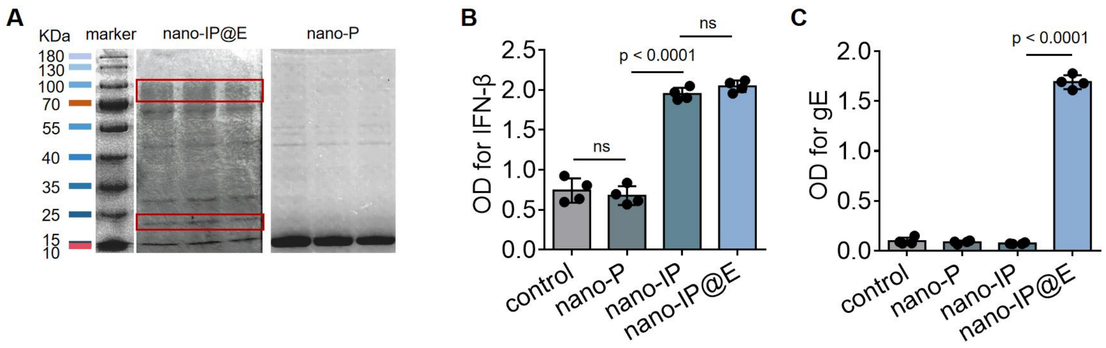

Figure S1. Protein characterization of nano-IP@E. (A) SDS-PAGE analysis of nano-IP@E and nano-P, with molecular weight markers shown on the left. Red boxes indicate the expected positions of target proteins: the upper box corresponds to VZV gE, and the lower box corresponds to IFN-β. ELISA quantification of (B) IFN-β and (C) VZV gE expression in control, nano-P, nano-IP, and nano-IP@E samples (n = 4 per group). Data are presented as mean ± SD. ns: not significant.

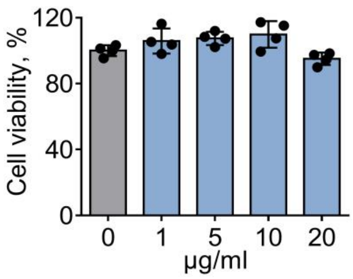

Figure S2. Cell viability of DCs after nano-IP@E treatment at various concentrations (n = 4 per group). Data are presented as mean ± SD.

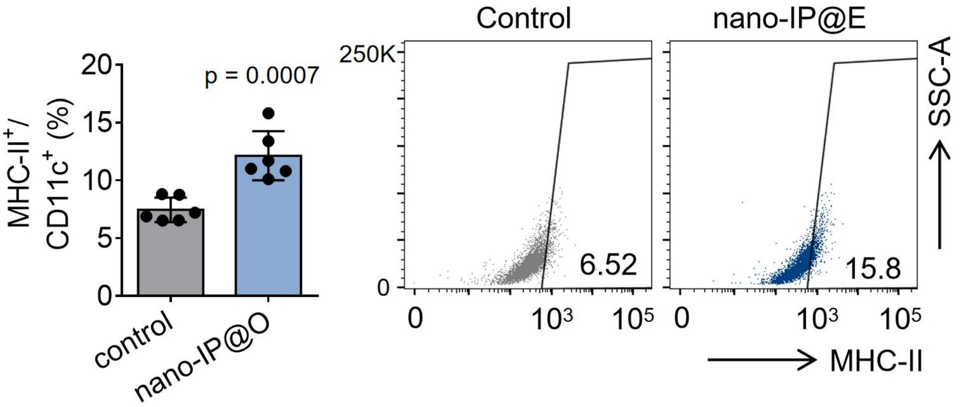

Figure S3. Flow cytometry analysis of MHC-II^{+}CD11c^{+} DCs in control and nano-IP@E treated groups, shown as percentage (left) (n = 6 per group) and representative density plots (right). Data are presented as mean ± SD.

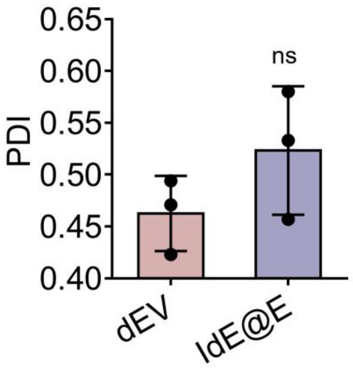

Figure S4. PDI values were measured to assess size distribution uniformity of dEV and IdE@E (n = 3 per group). Data are presented as mean ± SD. ns: not significant.

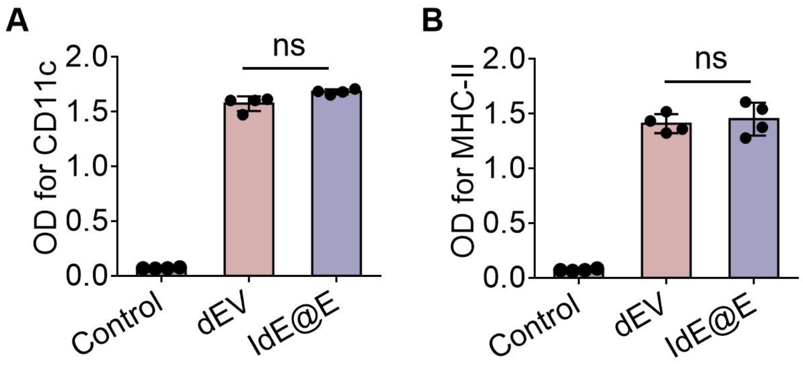

Figure S5. ELISA analysis of surface markers on dEV and IdE@E, including (A) CD11c and (B) MHC-II expression in control (PBS), dEV, and IdE@E samples (n = 4 per group). Data are presented as mean ± SD. ns: not significant.

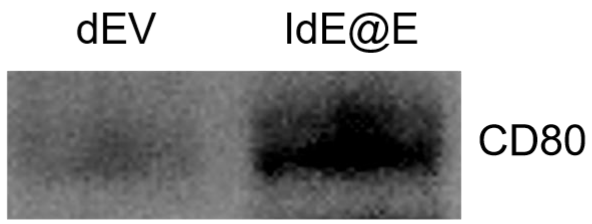

Figure S6. Western blot analysis of CD80 expression in dEV and IdE@E samples.

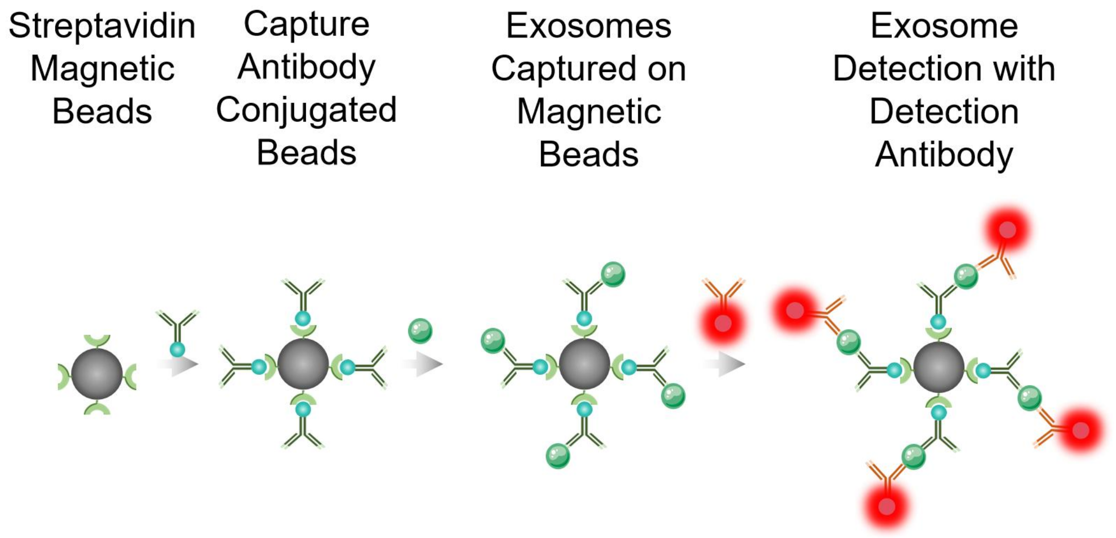

Figure S7. Schematic of magnetic bead detection method for exosome surface markers. This four-step process utilizes streptavidin-coated magnetic beads conjugated with capture antibodies specific to exosomal surface antigens. Exosomes in the sample bind to these antibodies, becoming immobilized on the magnetic beads. Subsequently, fluorescently labeled detection antibodies are introduced, binding to additional epitopes on the captured exosomes. This method enables quantitative analysis of exosomes through measurement of the fluorescence signal, with the flexibility to target various exosomal markers by altering the antibody pairs. The technique offers a highly specific and efficient approach for exosome characterization and quantification, potentially applicable in both research and clinical contexts, particularly for the analysis of extracellular vesicle-associated biomarkers.

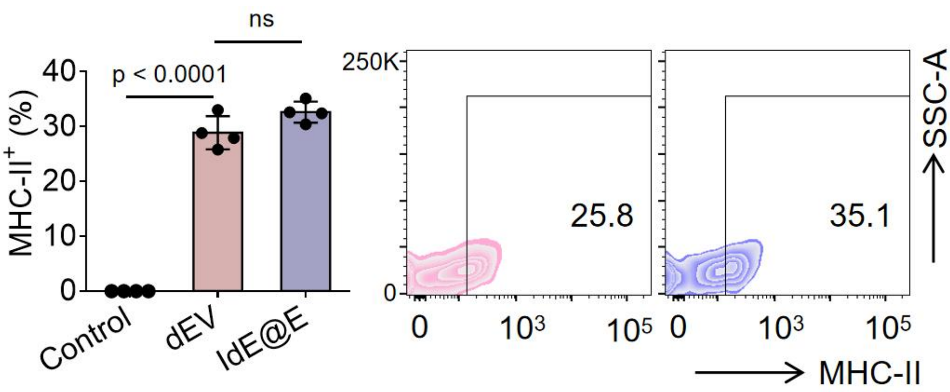

Figure S8. Flow cytometry analysis of MHC-II expression on dEV and IdE@E, shown as percentage of positive cells (left) ($n = 4$ per group) and representative density plots (right). Data are presented as mean ± SD. ns: not significant.

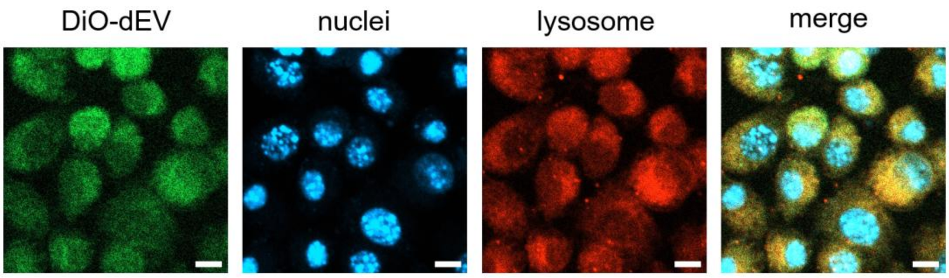

Figure S9. Confocal microscopy images showing cellular localization of DiO-labeled dEV (green), nuclei (blue), and lysosomes (red), with merged image. Scale bar = 10 μm.

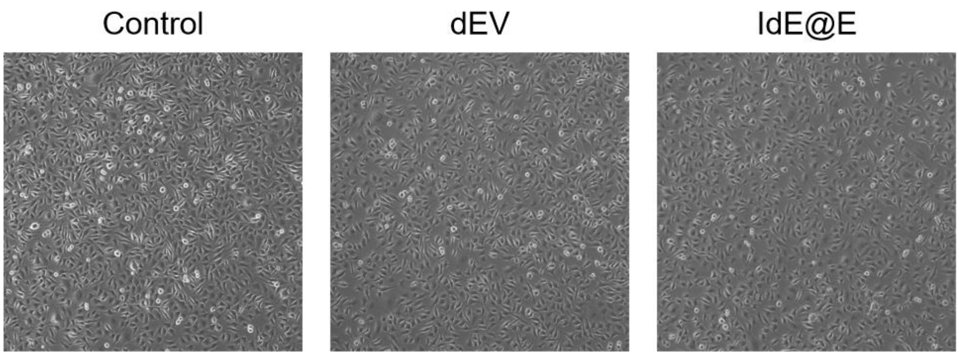

Figure S10. Bright-field microscopy images of L929 cells after various treatments.

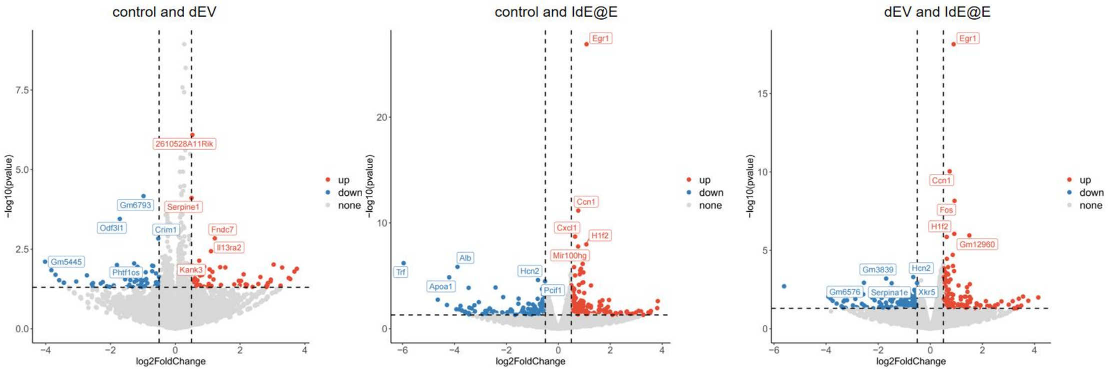

Figure S11. Differential gene expression analysis among all three groups. Volcano plot showing differentially expressed genes in dEV-treated cells compared to control. Red dots indicate significantly upregulated genes, blue dots indicate significantly downregulated genes, and grey dots indicate non-significant changes (|log2FoldChange| > 1, adjusted p-value < 0.05). Selected differentially expressed genes are labeled.

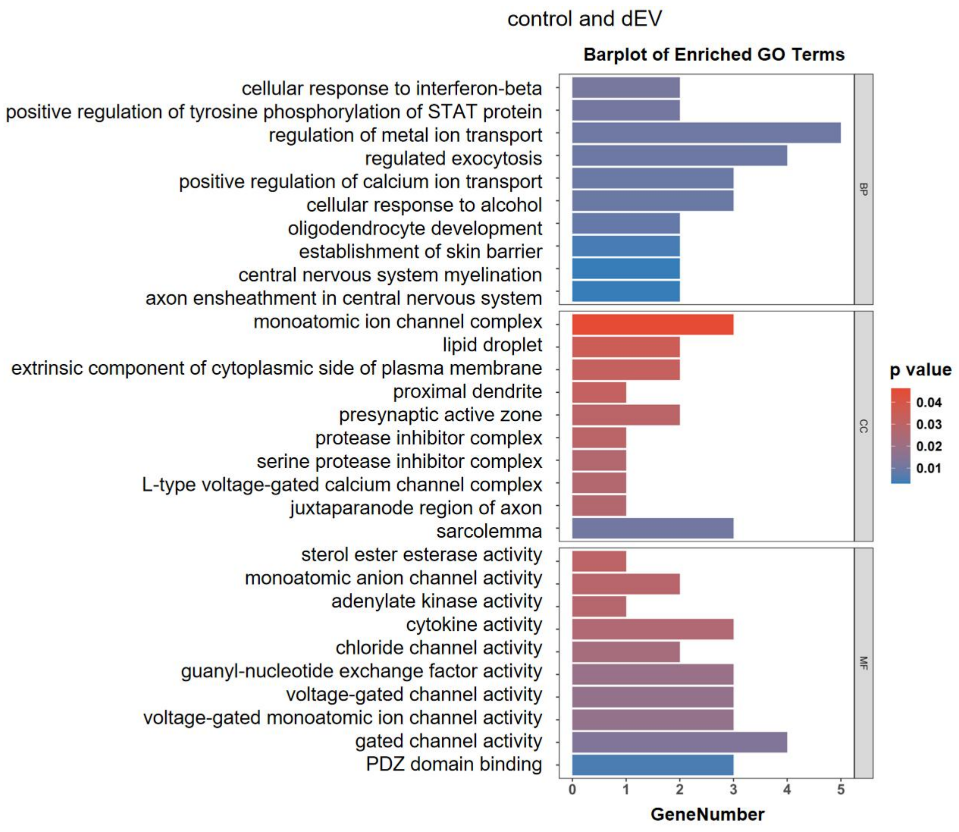

Figure S12. GO enrichment analysis of differentially expressed genes between control and dEV-treated cells. Bar plot showing significantly enriched GO terms categorized into biological processes (BP), cellular components (CC), and molecular functions (MF). Bar length represents the number of genes enriched in each term, and color indicates p-value significance (scale from 0.01 to 0.04). Terms include cellular responses, ion transport regulation, structural components, and various molecular activities.

control and dEV

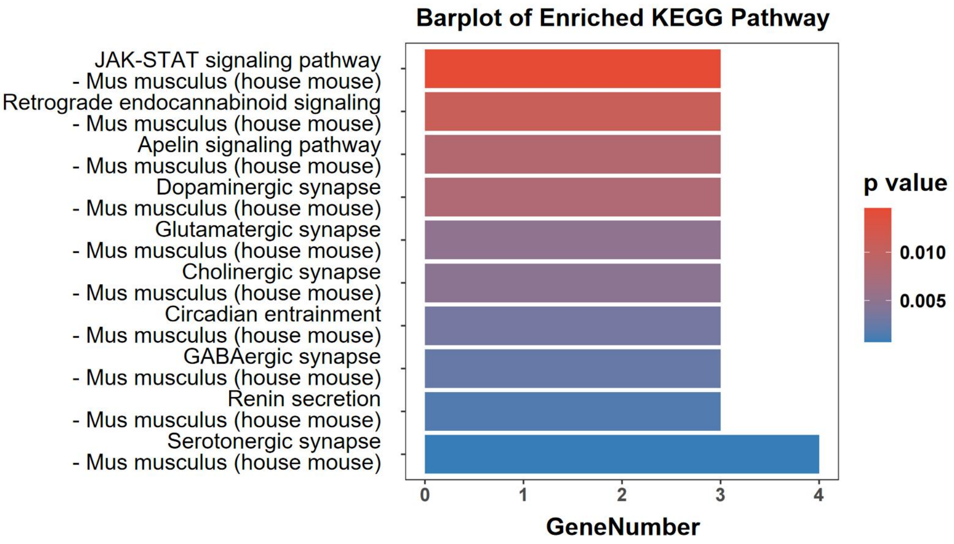

Figure S13. KEGG pathway enrichment analysis of differentially expressed genes between control and dEV-treated cells. Bar plot showing significantly enriched KEGG pathways in mouse (Mus musculus). Bar length represents the number of genes enriched in each pathway, and color indicates p-value significance (scale from 0.005 to 0.010). Enriched pathways include signaling cascades (JAK-STAT, endocannabinoid, apelin) and various synaptic pathways.

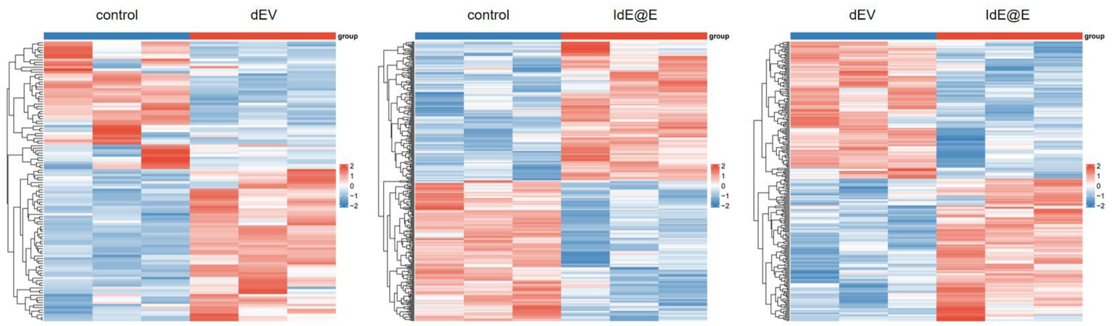

Figure S14. Heatmap visualization of differential gene expression patterns across experimental groups. Hierarchical clustering of differentially expressed genes shown for three comparisons: control vs dEV (left), control vs IdE@E (middle), and dEV vs IdE@E (right). Color scale represents normalized expression values, with red indicating higher expression and blue indicating lower expression (z-score range: -2 to 2). Each row represents a gene, and each column represents a sample within the indicated groups.

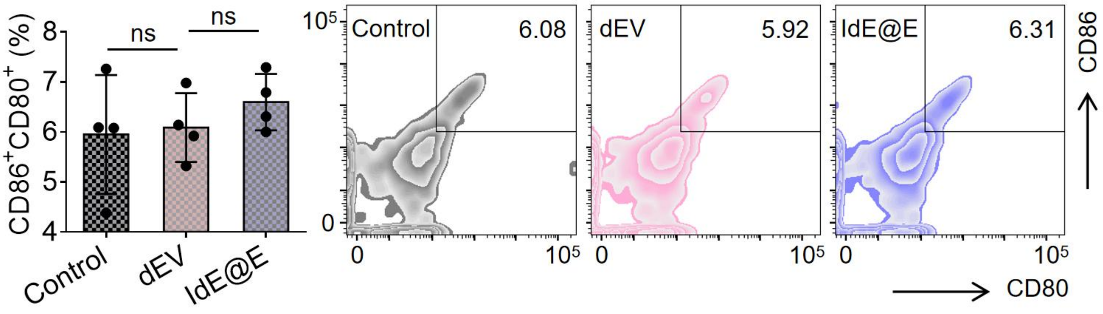

Figure S15. Flow cytometry analysis of $CD80^{+}CD86^{+}$ expression in dendritic cells after different treatments. Left: Quantification of $CD80^{+}CD86^{+}$ percentage across control, dEV, and IdE@E treated groups (n = 4 per group). Right: Representative flow cytometry density plots showing CD80 and CD86 expression. Data are presented as mean ± SD. ns: not significant.

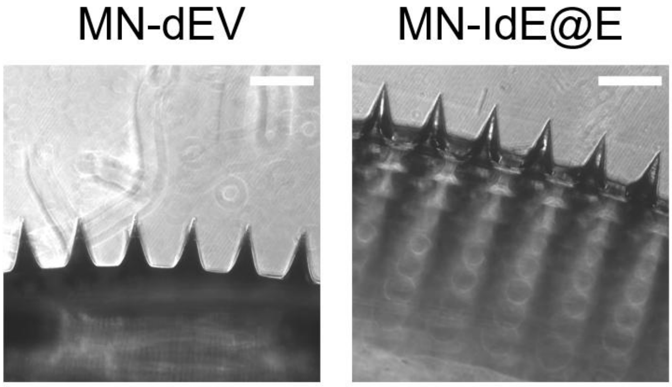

Figure S16. Side-view optical microscopy images of microneedle arrays. Scale bar = 500 μm.

MN-DiO-IdE@E

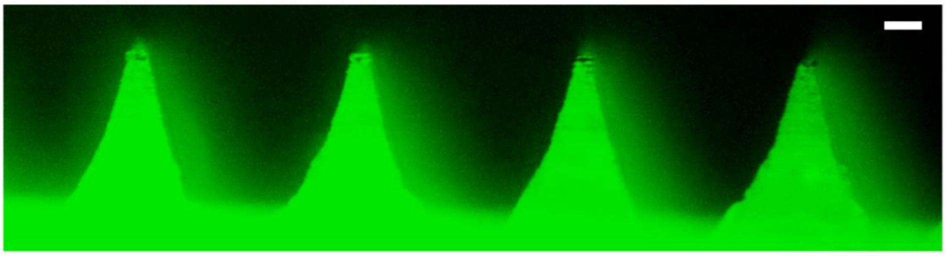

Figure S17. Side-view fluorescence microscopy image of DiO-labeled IdE@E distribution in microneedles. Green fluorescence shows the uniform distribution of DiO-labeled IdE@E within the microneedle structure, demonstrating successful loading of the cargo into the microneedle array. Scale bar = 100 μm.

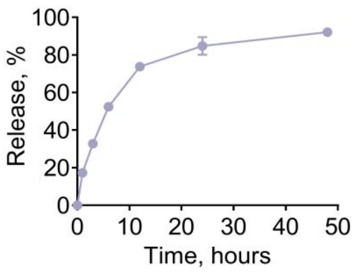

Figure S18. *In vitro* release profile of IdE@E from dissolvable microneedles (n = 4 per group). Data are presented as mean ± SD.

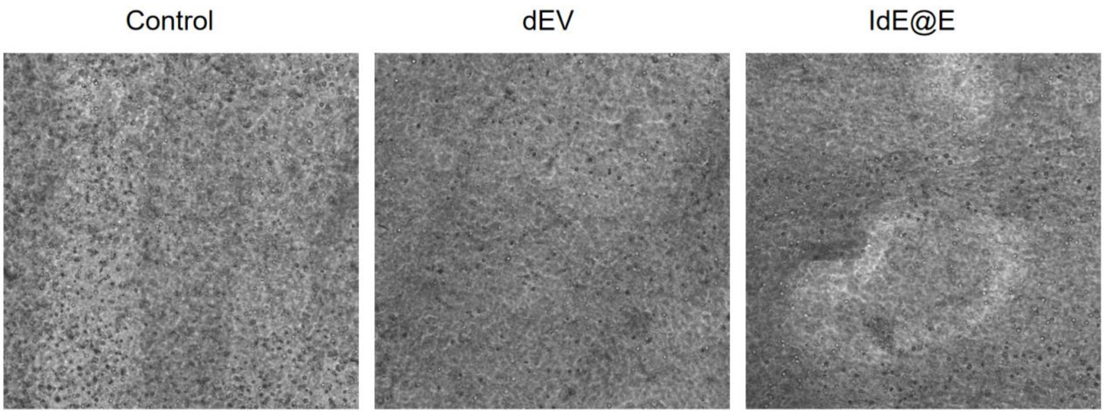

Figure S19. Optical microscopy images of fibroblasts in skin organoid model after various treatments.

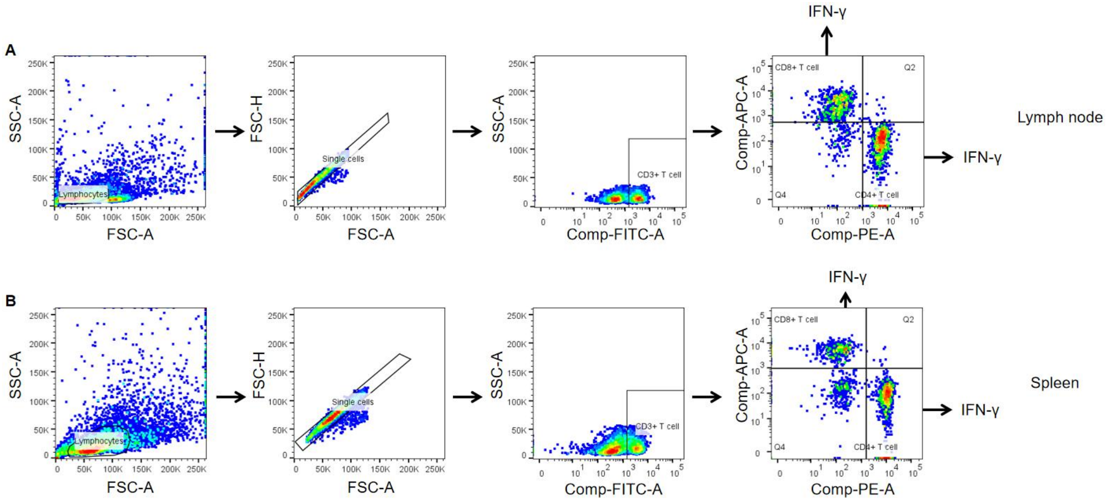

Figure S20. Gating strategy for flow cytometry analysis of T cells in key lymphoid organs. (A) Gating strategy for lymph node T cells. (B) Gating strategy for spleen T cells. This figure demonstrates the critical gating approach used in the core experiment of this study, which forms the foundation for our main findings on T cell responses.

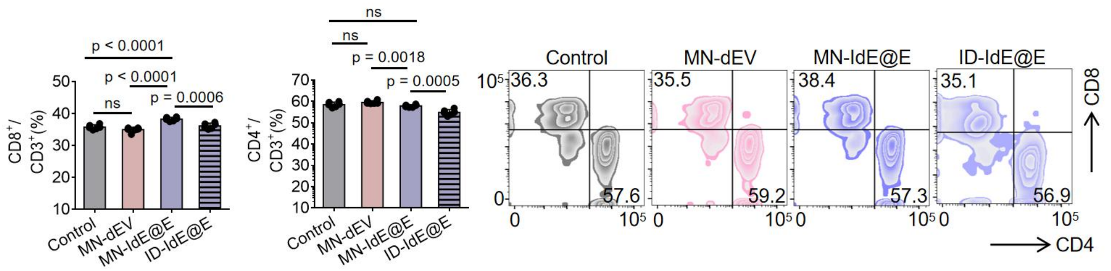

Figure S21. Flow cytometry analysis of $CD4^+$ and $CD8^+$ T cell proportions in lymph nodes after various treatments. Quantification of $CD8^+$ T cells and $CD4^+$ T cells as a percentage of $CD3^+$ T cells (n = 6 per group). Right: Representative flow cytometry plots showing $CD4^+$ and $CD8^+$ T cell distributions for each treatment group. Data are presented as mean ± SD. ns: not significant.

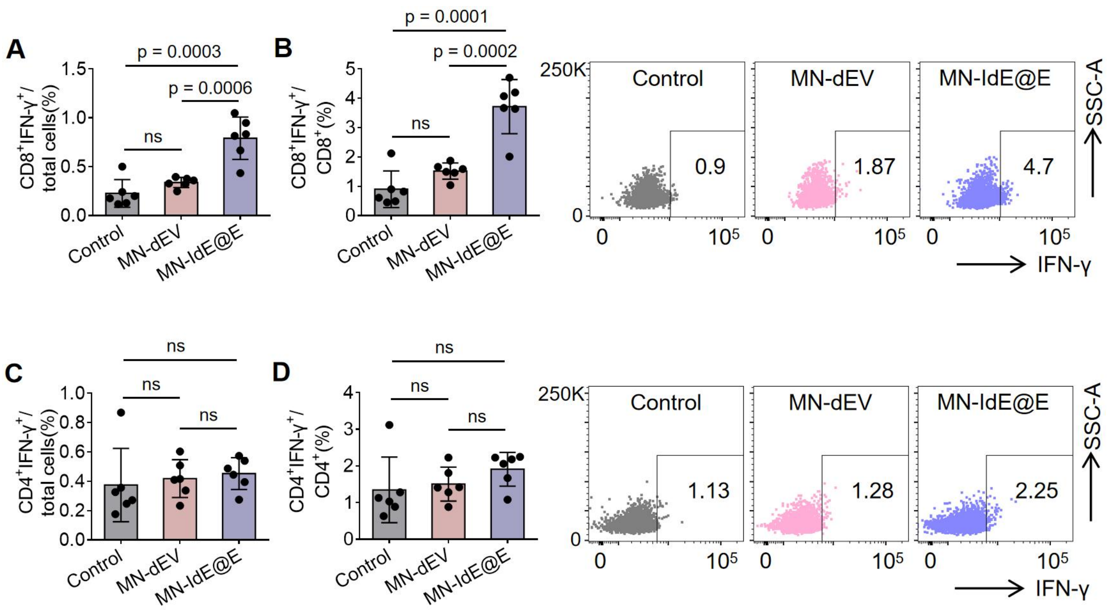

Figure S22. Analysis of IFN-γ-producing T cells in lymph nodes 24 days post-immunization. (A-B) Percentage of $CD8^+IFN\text{-}\gamma^+$ cells among total cells (A) and $CD8^+$ T cells (B) in lymph nodes (n = 6 per group), with representative flow cytometry plots (right). (C-D) Percentage of $CD4^+IFN\text{-}\gamma^+$ cells among total cells (C) and $CD4^+$ T cells (D) in lymph nodes (n = 6 per group), with representative flow cytometry plots (right). Data are presented as mean ± SD. ns: not significant.

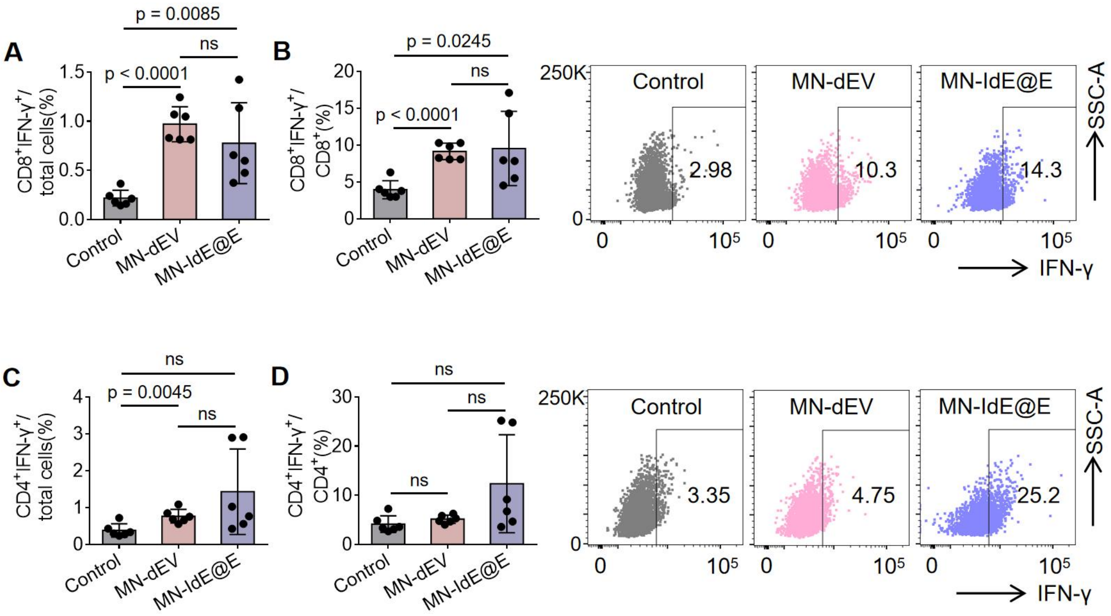

Figure S23. Analysis of IFN-γ-producing T cells in spleens 24 days post-immunization. (A-B) Percentage of $CD8^{+}IFN\text{-}\gamma^{+}$ cells among total cells (A) and $CD8^{+}$ T cells (B) in spleens (n = 6 per group), with representative flow cytometry plots (right). (C-D) Percentage of $CD4^{+}IFN\text{-}\gamma^{+}$ cells among total cells (C) and $CD4^{+}$ T cells (D) in spleens (n = 6 per group), with representative flow cytometry plots (right). Data are presented as mean ± SD. ns: not significant.

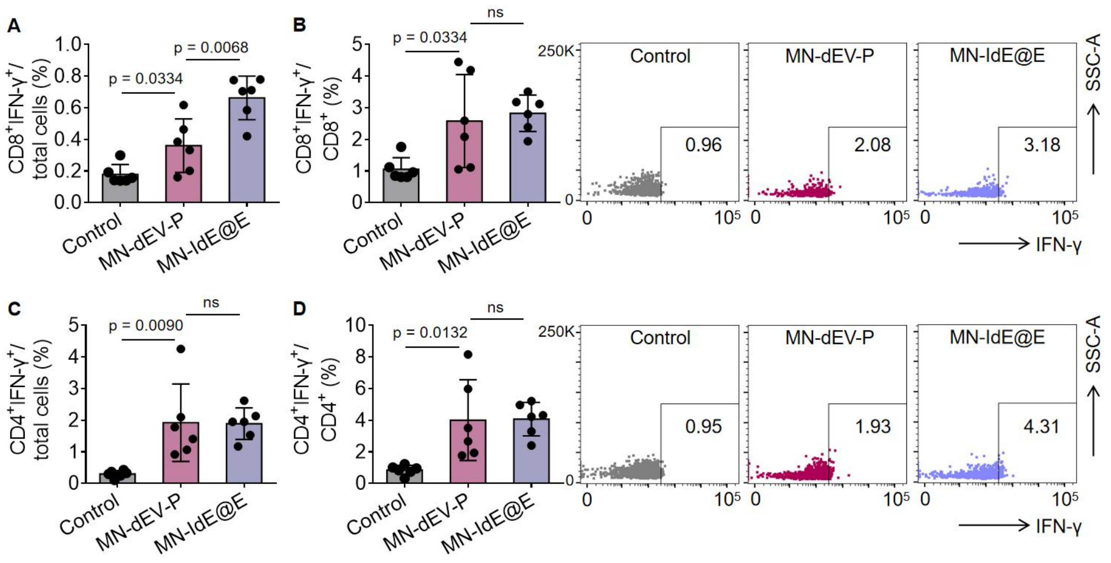

Figure S24. A supplementary control group (dEV-P) showing T cell responses in lymph nodes 16 days post-immunization. (A-B) Percentage of CD8$^+$IFN-γ$^+$ cells among total cells (A) and CD8$^+$ T cells (B) in lymph nodes (n = 6 per group), with representative flow cytometry plots (right). (C-D) Percentage of CD4$^+$IFN-γ$^+$ cells among total cells (C) and CD4$^+$ T cells (D) in lymph nodes (n = 6 per group), with representative flow cytometry plots (right). Data are presented as mean ± SD. ns: not significant.

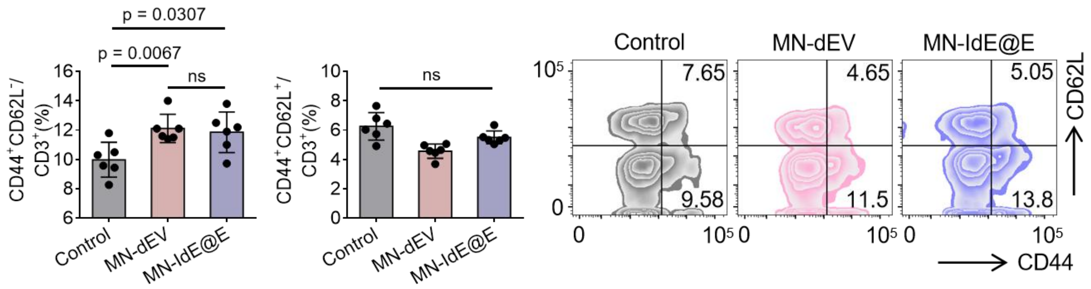

Figure S25. Analysis of memory T cells in lymph nodes 24 days post-immunization. Percentage of $CD44^{+}CD62L^{-}$ effector memory T cells and $CD44^{+}CD62L^{+}$ central memory T cells among $CD3^{+}$ T cells (n = 6 per group), with representative flow cytometry plots showing CD44 and CD62L expression patterns for control, MN-dEV, and MN-IdE@E groups. Data are presented as mean ± SD. ns: not significant.

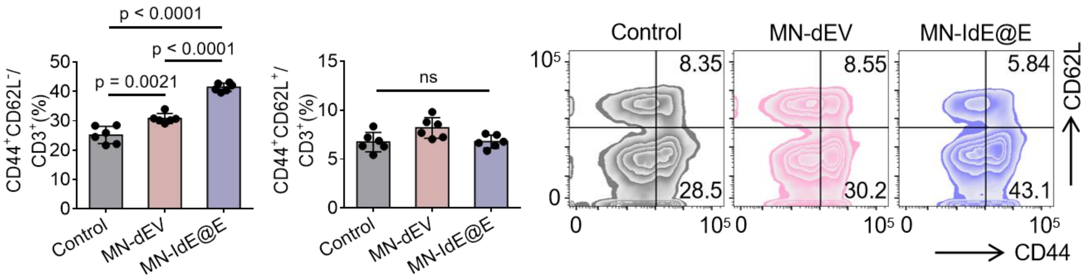

Figure S26. Analysis of memory T cells in spleens 24 days post-immunization. Percentage of $CD44^{+}CD62L^{-}$ effector memory T cells and $CD44^{+}CD62L^{+}$ central memory T cells among $CD3^{+}$ T cells (n = 6 per group), with representative flow cytometry plots showing CD44 and CD62L expression patterns for control, MN-dEV, and MN-IdE@E groups. Data are presented as mean ± SD. ns: not significant.

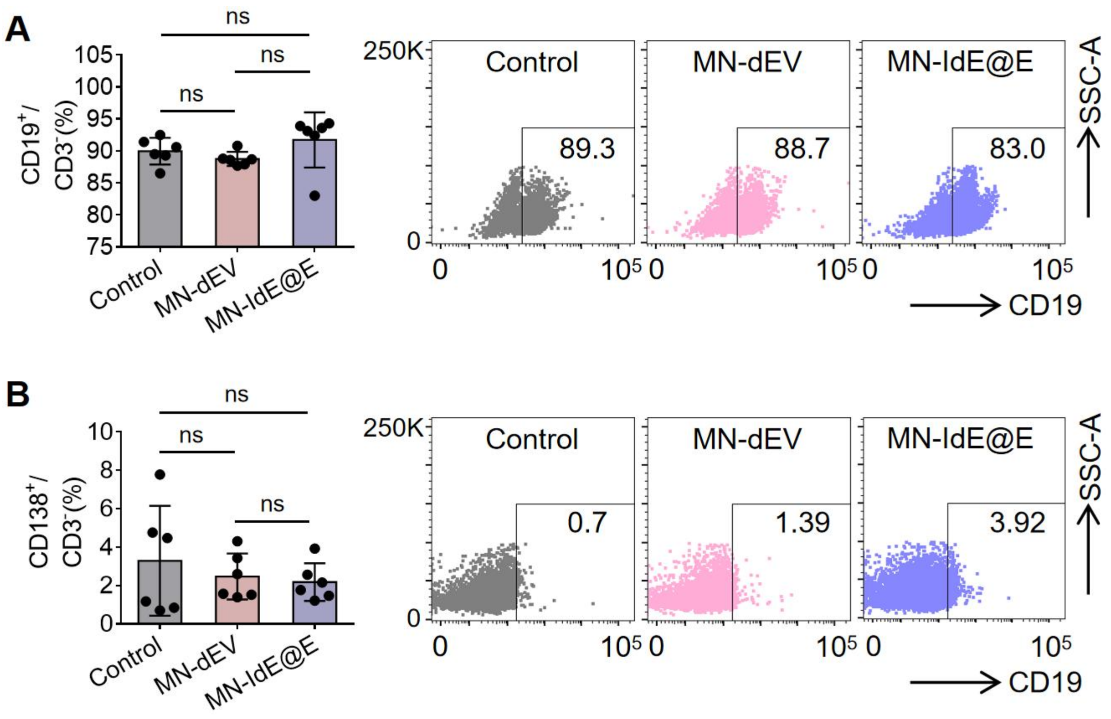

Figure S27. Analysis of B cell populations in lymph nodes 24 days post-immunization. Percentage of (A) $CD19^+$ cells and (B) $CD138^+$ cells among $CD3^-$ cells (n = 6 per group), with representative flow cytometry plots (right). Data are presented as mean ± SD. ns: not significant.

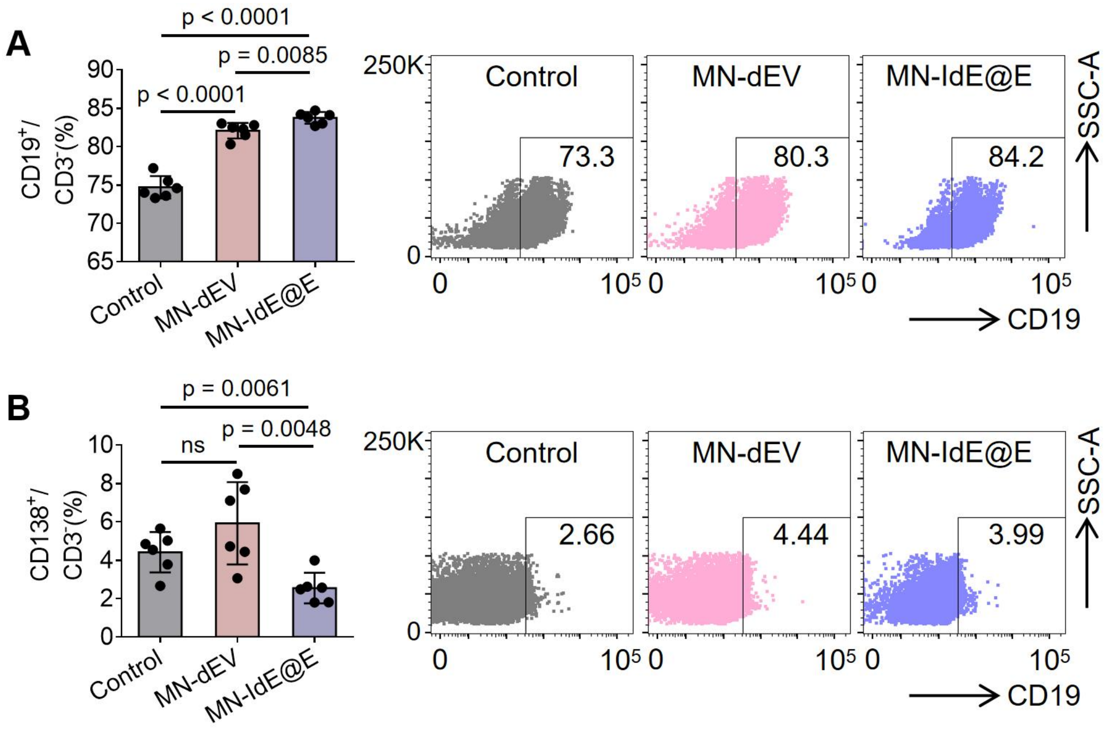

Figure S28. Analysis of B cell populations in spleens 24 days post-immunization. Percentage of (A) CD19^{+} cells and (B) CD138^{+} cells among CD3^{-} cells (n = 6 per group), with representative flow cytometry plots (right). Data are presented as mean ± SD. ns: not significant.

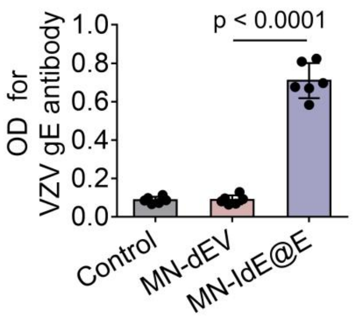

Figure S29. Serum VZV gE-specific IgG antibody levels after various treatments (n = 6 per group). Data are presented as mean ± SD.

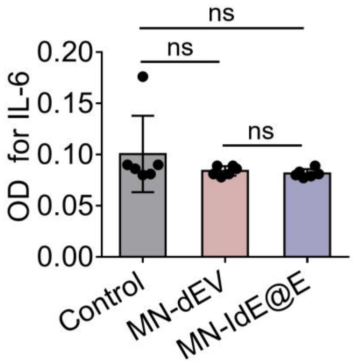

Figure S30. Serum IL-6 levels after various treatments (n = 6 per group). Data are presented as mean ± SD. ns: not significant.

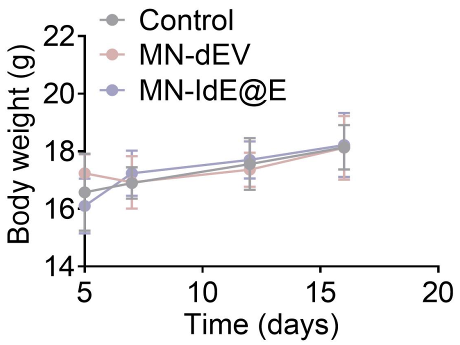

Figure S31. Body weight changes in mice over 16 days following immunization (n = 6 per group). Data are presented as mean ± SD.

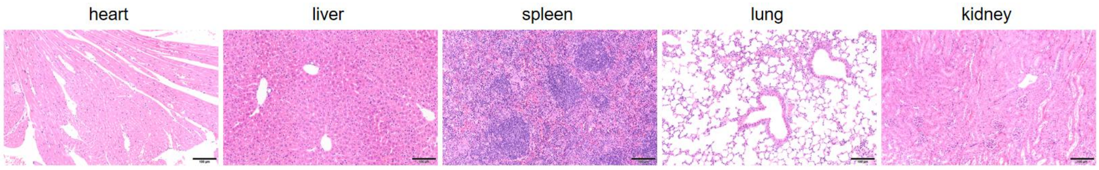

Figure S32. H&E stained tissue sections of major organs (heart, liver, spleen, lung, kidney) after MN-IdE@E treatment. Scale bar = 100 μm.
